# Biochemical Investigation of the Interaction of pICln, RioK1 and COPR5 with the PRMT5–MEP50 Complex

**DOI:** 10.1002/cbic.202100079

**Published:** 2021-03-31

**Authors:** Adrian Krzyzanowski, Raphael Gasper, Hélène Adihou, Peter 't Hart, Herbert Waldmann

**Affiliations:** ^1^ Department of Chemical Biology Max Planck Institute of Molecular Physiology Otto-Hahn-Strasse 11 44227 Dortmund Germany; ^2^ Faculty of Chemistry Chemical Biology Technical University Dortmund Otto-Hahn-Strasse 6 44221 Dortmund Germany; ^3^ Crystallography and Biophysics Unit Max-Planck-Institute of Molecular Physiology Otto-Hahn-Strasse 11 44227 Dortmund Germany; ^4^ AstraZeneca MPI Satellite Unit Department of Chemical Biology Max Planck Institute of Molecular Physiology Otto-Hahn-Strasse 11 44227 Dortmund Germany; ^5^ Medicinal Chemistry Research and Early Development Cardiovascular, Renal and Metabolism, BioPharmaceuticals R&D AstraZeneca Otto-Hahn-Strasse 11 44227 Gothenburg Sweden; ^6^ Chemical Genomics Centre of the Max Planck Society Max Planck Institute of Molecular Physiology Otto-Hahn-Strasse 11 44227 Dortmund Germany

**Keywords:** interfaces, peptides, protein–protein interactions, proteins

## Abstract

The PRMT5–MEP50 methyltransferase complex plays a key role in various cancers and is regulated by different protein–protein interactions. Several proteins have been reported to act as adaptor proteins that recruit substrate proteins to the active site of PRMT5 for the methylation of arginine residues. To define the interaction between these adaptor proteins and PRMT5, we employed peptide truncation and mutation studies and prepared truncated protein constructs. We report the characterisation of the interface between the TIM barrel of PRMT5 and the adaptor proteins pICln, RioK1 and COPR5, and identify the consensus amino acid sequence GQF[D/E]DA[E/D] involved in binding. Protein crystallography revealed that the RioK1 derived peptide interacts with a novel PPI site.

## Graphical abstract

**Determination of a PPI interface**: The adaptor protein binding site of PRMT5 has been identified and studied by using synthetic peptides. A cocrystal structure of the PRMT5 TIM‐barrel domain and a RioK1‐derived peptide highlights the site of interaction.

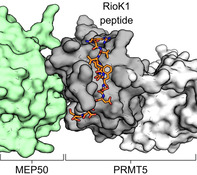

## Introduction

Methylation of terminal arginine nitrogens by protein arginine methyltransferases (PRMTs) plays important roles in various cellular processes, including the establishment of cancer.^[1]^ Symmetric methylation of both arginine nitrogens is mainly catalysed by PRMT5, which methylates histones H2A, H3 and H4 and various non‐histone targets.^[2]^ Target selection depends on adaptor proteins which bind to PRMT5 and recruit specific substrates to the PRMT5 active site.^[2a]^ Adaptor proteins include MEP50 (WDR77; which plays an important role in histone methylation),^[3]^ pICln (which recruits Sm proteins),^[4]^ RioK1 (which recruits nucleolin)^[5]^ and COPR5 (which recruits histone H4).^[6]^ pICln and RioK1 bind mutually exclusive to PRMT5 and compete for the same binding site, but are not competitive with MEP50.^[5]^

**Figure 1:**
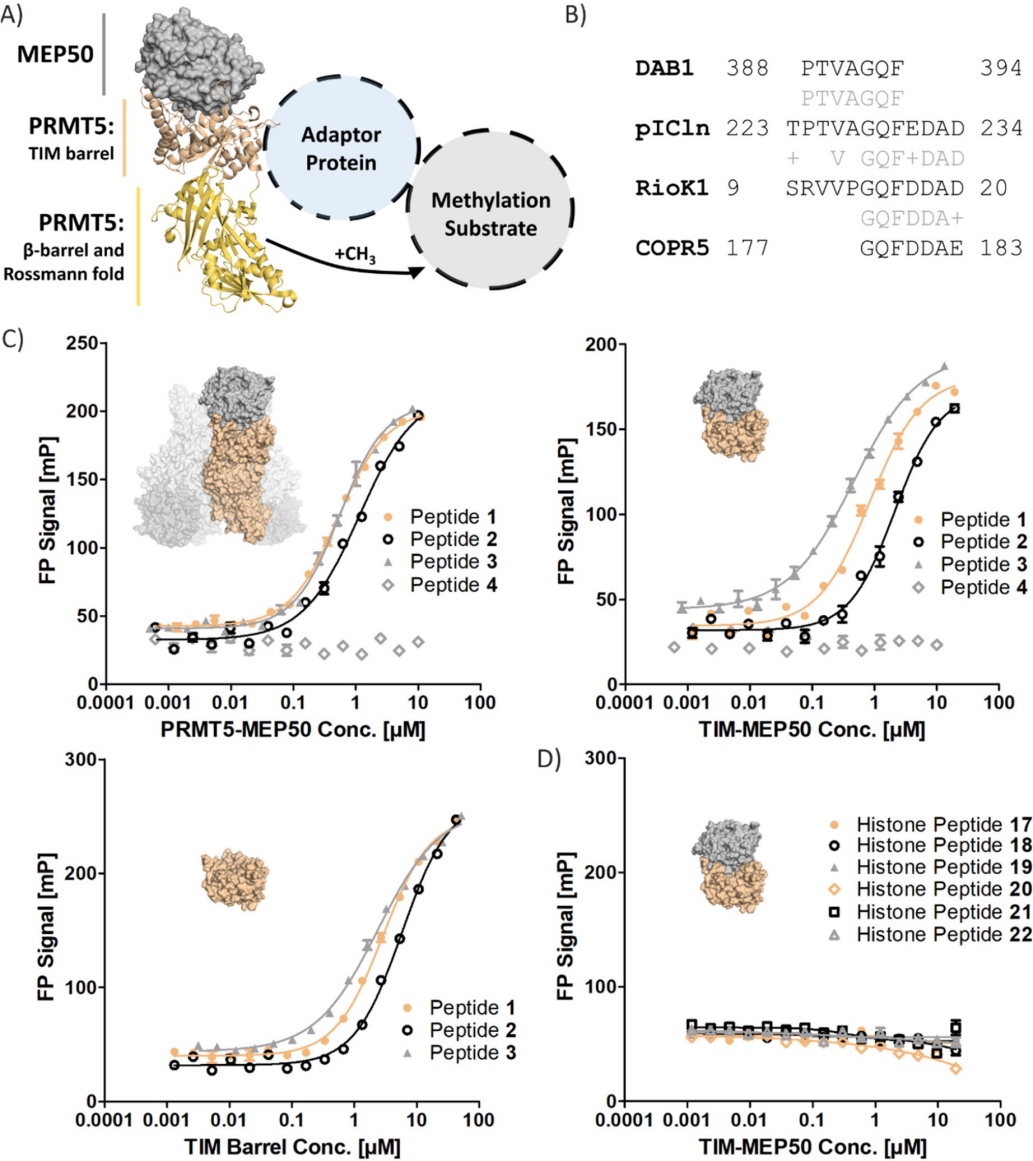
Interaction between PRMT5 and its adaptor proteins. A) Schematic representation of the PRMT5–adaptor protein complexes with their methylation substrate. The PRMT5–MEP50 complex was visualised based on the PDB structure 4GQB. B) BLAST alignments for pICln, RioK1, COPR5 and DAB1. C) Results of the fluorescence polarization measurements for interaction of the identified peptide sequences with the full PRMT5–MEP50 complex, truncated TIM–MEP50 and isolated TIM barrel domain (*n*=3). D) Results of fluorescence polarisation assays for histone tail peptides and the truncated protein complex TIM–MEP50 (*n*=3).

PRMT5 contains three domains, that is, the catalytic site‐containing Rossmann fold, a β‐barrel domain used for dimerization, and the TIM barrel, which acts as interaction site for MEP50 (Figure 1A).[^3a^, ^3c^] PRMT5 and MEP50 form a hetero‐octameric complex whose structure has been determined,^[3a]^ and structural information on the interaction between PRMT5 and the other adaptor proteins has only very recently been described in preliminary form.^[7]^ Overexpression of PRMT5 is frequently observed in cancer which makes the protein an intensively investigated anticancer target, and active‐site directed inhibitors have reached clinical trials.^[8]^ Further study of the PRMT5 PPIs and binding partners is important for a better understanding of its regulation.

Herein, we describe the use of synthetic peptides to study the PRMT5–adaptor protein interaction by biochemical methods and protein crystallography. An adaptor protein consensus sequence was derived by sequence alignment and synthetic peptides derived from this consensus were prepared. The site of interaction was initially determined by truncations of PRMT5 itself and measuring the affinity for synthetic adaptor protein peptides. Truncation and alanine scanning of the adaptor protein peptides allowed identification of the residues contributing to the interaction. A co‐crystal structure of a RioK1 derived peptide with the PRMT5 TIM barrel domain confirmed the results of our mutation and truncation studies. Our findings are in agreement with the recent preliminary report by Sellers et al.^[7]^

## Results and Discussion

Intrigued by the report of Guderian et al., noting mutually exclusive binding of pICln and RioK1 to PRMT5, we aimed to identify common features between the two proteins to identify a potential interaction domain.^[5]^ Due to the very limited structural information available for pICln and RioK1, at the time of initiating the project, we focused on the analysis and comparison of the protein sequences. Protein BLAST alignment identified significant similarity in sequences between the C‐terminal region of pICln (TPTVAGQFEDAD, residues 223–234) and the N‐terminal region of RioK1 (SRVVPGQFDDAD, residues 9–20) as shown in Figure 1B.^[9]^ The identified sequences were then used for a global similarity search among available *Homo sapiens* protein data, resulting in the identification of two new sequence matches: COPR5 (GQFDDAE, residues 177–183) and DAB1 (PTVAGQF, residues 388–394; Figure 1B).

Notably, the identified sequences of RioK1, pICln and COPR5 were part of the protein regions, previously proposed to be responsible for binding to PRMT5 (Figure 1C). The C‐terminal region of pICln containing the acid domain AD3 (residues 230–237), and regions in the PH domain (residues 1–134) were shown to mediate the PPI with PRMT5.[^4b^, ^10^] Moreover, the N‐terminal fragment of RioK1 (residues 1–120) and the C‐terminal fragment of COPR5 (residues 141–184) were previously reported as the main contributors to the interactions with the methyltransferase.[^5^, ^6^] We noted that in all cases the common GQF[D/E]DA[E/D] sequence occurred in the regions involved in the PPIs with PRMT5. The same sequences were identified by Sellers et al. by applying BioGrid analysis.^[9]^

Fluorescently labelled peptides, **1**, **2**, **3** and **4** (Table 1), corresponding to the determined sequence matches for RioK1, pICln, COPR5 and DAB1, respectively, were synthesised to be tested for binding by using a fluorescence polarisation (FP) assay. An untagged, native, human PRMT5–MEP50 complex was expressed and tested for activity using the MTAse Glo assay (Figure S10 in the Supporting Information). When tested for binding we observed potent interaction of **1** (*K*_D_ 0.52 μM), **2** (*K*_D_ 1.14 μM) and **3** (*K*_D_ 0.55 μM), but not **4** (Figure 1C top left and Table 1).

**Table 1:** Sequences and *K*_D_ values for the initial RioK1, pICln, COPR5 and DAB1 derived, fluorescently labelled peptides **1**–**4**.

Peptide	Sequence	*K*_D_ value [μM]^[a]^		
		Full‐length PRMT5–MEP50	TIM–MEP50	TIM barrel
**1** (RioK1)	Fitc‐O2Oc‐SRVVP**GQFDDAD**‐NH_2_	0.52±0.04	0.84±0.15	2.8±0.07
**2** (pICln)	Fitc‐O2Oc‐TPTVA**GQFEDAD**‐NH_2_	1.14±0.04	2.1±0.15	5.8±0.11
**3** (COPR5)	Fitc‐O2Oc‐MVFET**GQFDDAE**D‐NH_2_	0.55±0.12	0.46±0.00	2.4±0.32
**4** (DAB1)	Fitc‐O2Oc‐PTVAGQF‐NH_2_	n.b.	n.b.	n.d.

[a] As determined with FP (*n*=3). n.b.: no binding, n.d.: not determined.

In order to identify the protein domain responsible for the interaction with the peptides, a truncated PRMT5 protein (residues 1–292), corresponding to the TIM barrel domain, was either expressed alone or co‐expressed as a complex with MEP50 (Figure 1C). The truncation constructs lack key residues required for higher order oligomer formation and are therefore expected to exist as monomers which was confirmed by mass photometry (Figure S8).[^3a^, ^11^]

FP analysis of compounds **1**–**3** gave similar K_D_ values for all three tested protein constructs (Figure 1C and Table 1). These results clearly indicate that the peptides interact with the TIM barrel domain of PRMT5. The lower binding affinity to the isolated TIM barrel domain in comparison with the complexes containing MEP50, may be due to a potential loss of the stabilising effect of MEP50 on PRMT5. The labelled peptides did not show unspecific binding to BSA and GST (Figure S3 and S4). Compound **4** did not have affinity for any of the protein constructs indicating the negatively charged C‐terminal section is critical for binding.

In order to further define the requirements for pICln/RioK1/COPR5‐PRMT5 binding, we synthesised a series of RioK1 and pICln derived peptides (Table 2) to determine the shortest sequence necessary for effective interaction. Any truncation on the C‐terminal side (compare compounds **5**, **6** and **14** to **1**) led to a significant loss of affinity. To explore N‐terminal truncation, C‐terminally labelled **7** was synthesised which bound with a similar affinity as peptide **1**. Truncating the N‐terminus showed that loss of the first three residues improved the binding (compound **8**) but any further truncation led to a substantial loss of affinity (compounds **9** and **10**). Compound **8**, with the nine amino acid sequence VPGQFDDAD, constituted the smallest but still efficiently binding peptide fragment allowing for the interaction with PRMT5 (*K*_D_=208 nM). Extensions of either the N‐ or C‐termini led to improvements over peptide **1** but not in comparison to **8**. Peptides derived from pICln showed similar trends where deletion of the negatively charged C‐terminal section resulted in loss of binding (compare compound **14** to **2**) while extension led to an increase (compare **15** to **2**). In contrast to the RioK1 derived peptides extension on the N‐terminus of the pICln peptide resulted in a twofold decrease in affinity (compare **16** to **2**).

**Table 2:** Structures and K_D_ values of truncated and extended pICln, RioK1 and COPR5 derived, fluorescently labelled linear peptides.

Peptide	Sequence	*K*_D_ [nM]^[a]^
**1** (RioK1)	Fitc‐O2Oc‐SRVVP**GQFDDAD**‐NH_2_	522±45
**2** (pICln)	Fitc‐O2Oc‐TPTVA**GQFEDAD**‐NH_2_	1145±38
**3** (COPR5)	Fitc‐O2Oc‐MVFET**GQFDDAE**D‐NH_2_	549±119
**4** (DAB1)	Fitc‐O2Oc‐PTVAGQF‐NH_2_	n.b.
**5**	Fitc‐O2Oc‐SRVVPGQFDD‐NH_2_	>5000
**6**	Fitc‐O2Oc‐SRVVPGQF‐NH_2_	n.b.
**7**	Ac‐SRVVPGQFDDADK(O2Oc‐Fitc)‐NH_2_	716±36
**8**	Ac‐VPGQFDDADK(O2Oc‐Fitc)‐NH_2_	**208±74**
**9**	Ac‐GQFDDADK(O2Oc‐Fitc)‐NH_2_	>5000
**10**	Ac‐FDDADK(O2Oc‐Fitc)‐NH_2_	n.b.
**11**	Fitc‐O2Oc‐SRVVPGQFDDADSSD‐NH_2_	295±11
**12**	Fitc‐O2Oc‐LLMSRVVPGQFDDAD‐NH_2_	192±26
**13**	Fitc‐O2Oc‐LLMSRVVPGQFDDADSSD‐NH_2_	279±41
**14**	Fitc‐O2Oc‐TPTVAGQF‐NH_2_	n.b.
**15**	Fitc‐O2Oc‐TPTVAGQFEDADVDH‐NH_2_	520±40
**16**	Fitc‐O2Oc‐VDTTPTVAGQFEDAD‐NH_2_	2378±817
**17** (H2A)	Fitc‐O2Oc‐SGRGKQGGKARAKAKTRSSRA‐NH_2_	n.b.
**18** (H4)	Fitc‐O2Oc‐SGRGKGGKGLGKGGAKRHRKV‐NH_2_	n.b.
**19** (H3)	Fitc‐O2Oc‐ARTKQTARKSTGGKAPRKQLATKAARKSA‐NH_2_	n.b.
**20** (H2A)	Ac‐SGRGKQGGKARAKAKTRSSRA−O2Oc−K(Fitc)‐NH_2_	n.b.
**21** (H4)	Ac‐SGRGKGGKGLGKGGAKRHRKV−O2Oc−K(Fitc)‐NH_2_	n.b.
**22** (H3)	Ac‐ARTKQTARKSTGGKAPRKQLATKAARKSA−O2Oc−K(Fitc)‐NH_2_	n.b.

[a] For peptide **1**–**16** determined with FP using the native PRMT5–MEP50 complex (*n*=3). For peptides **17**–**22** determined with FP using the TIM–MEP50 complex (*n*=3). n.b.: no binding.

To further explore the PPIs of PRMT5–MEP50 we investigated the interaction between histone tail peptides and the truncated TIM–MEP50 protein complex. We hypothesised that the histone tails could potentially bind to MEP50 in an analogous fashion to the H3‐WDR5 interactions, where the WD40‐repeat protein would act as a substrate presenter for PRMT5.^[12]^ Therefore, histone H2A, H3 and H4 tail peptides were synthesised (compounds **17**–**22**) and tested for binding to the TIM–MEP50 complex. No binding was observed in the performed FP assay (Figure 1D), strongly indicating that the histone tails do not interact with either MEP50 or the TIM barrel domain.

To confirm that RioK1, pICln and COPR5 bind to the same site, we used compound **11** as a tracer in a competitive FP experiment for binding to PRMT5–MEP50.^[5]^ All tested peptides (**23**–**25**) were able to compete with the fluorescently labelled **11**, indicating that all peptides share the same binding site (Table 3).

**Table 3:** Structures and IC_50_ values of pICln, RioK1 and COPR5 derived linear peptides and the results of the alanine scan for peptide **26**.

Peptide	Sequence	IC_50_ [μM]^[a]^
**23**	Ac‐SRVVPGQFDDADSSD‐NH_2_	1.5±0.4
**24**	Ac‐TPTVAGQFEDAD‐NH_2_	14.6±0.9
**25**	Ac‐MVFETGQFDDAED‐NH_2_	3.4±0.2
**26**	Ac‐SRVVPGQFDDAD‐NH_2_	6.0±0.7
**27**	Ac‐SRVVPGQFDDAA‐NH_2_	6.2±2.2
**28**	Ac‐SRVVPGQFDAAD‐NH_2_	97±17
**29**	Ac‐SRVVPGQFADAD‐NH_2_	6.4±0.3
**30**	Ac‐SRVVPGQADDAD‐NH_2_	>300
**31**	Ac‐SRVVPGAFDDAD‐NH_2_	>300
**32**	Ac‐SRVVPAQFDDAD‐NH_2_	39±8.5
**33**	Ac‐SRVVAGQFDDAD‐NH_2_	9.9±2.2
**34**	Ac‐SRVAPGQFDDAD‐NH_2_	14±3.4
**35**	Ac‐SRAVPGQFDDAD‐NH_2_	4.8±0.7
**36**	Ac‐SAVVPGQFDDAD‐NH_2_	2.8±0.5
**37**	Ac‐ARVVPGQFDDAD‐NH_2_	0.9±0.2
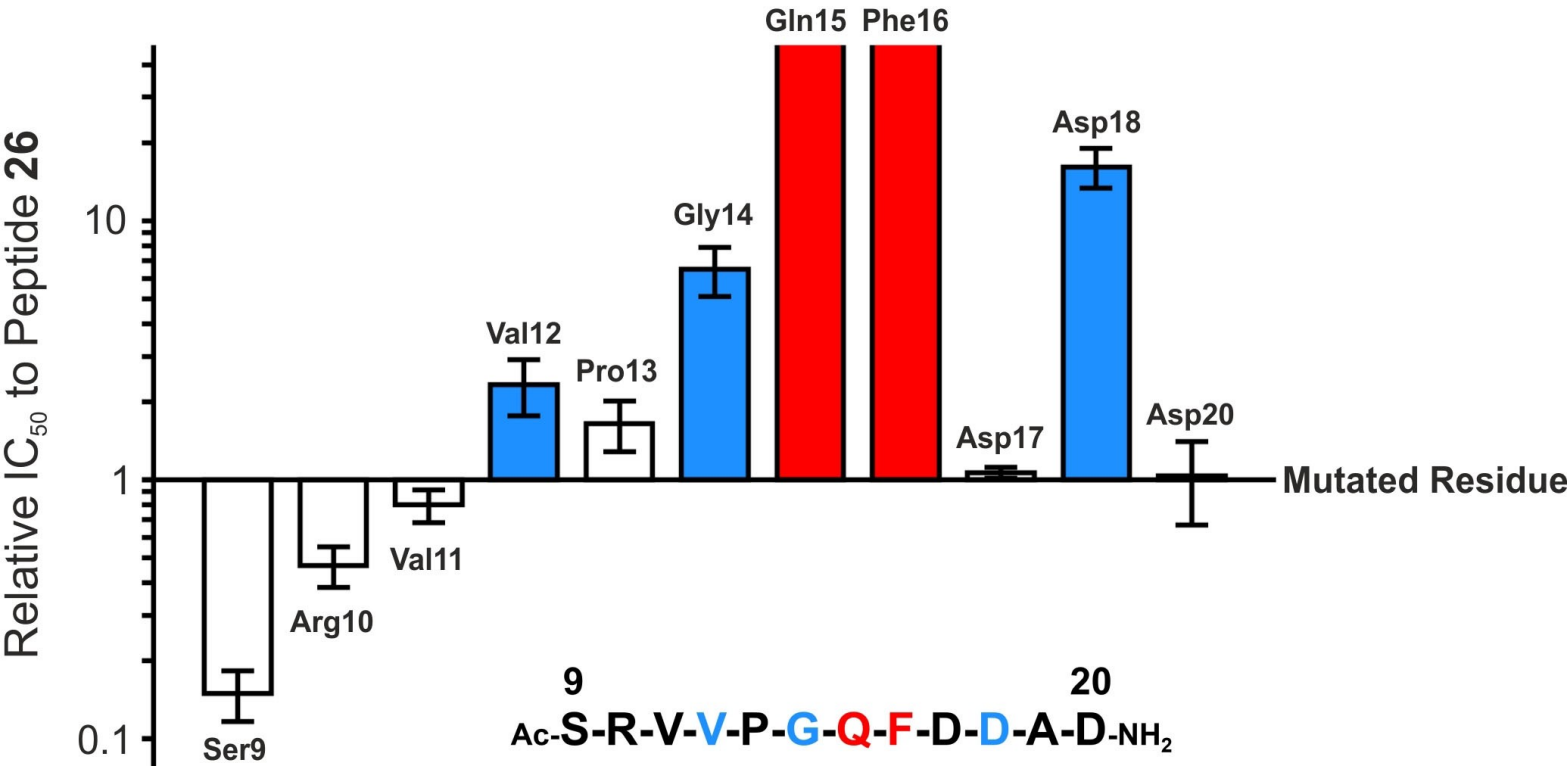		

[a] As determined with FP using the native PRMT5–MEP50 complex and compound **11** as a fluorescent tracer (*n*=3).

To identify hot spot residues which provide the main contribution to the interaction,^[13]^ we conducted an alanine amino acid scan for RioK1 derived peptide **26** (Table 3) which revealed that the side chains of Gln15 and Phe16 are indispensable for potent peptide binding to PRMT5 (peptides **31** and **30**). The side chains of Val12 and Asp18 also contributed to the interaction (compounds **34** and **28**), and substitution of Ala for Gly14 resulted in loss of affinity indicating that Gly is important for the correct conformation of the peptide (peptide **32**). Mutation of the residues in the SRV and AD region, had either no, or a moderately positive impact on the interaction with the protein (compounds **27**, **35**–**37**).

More insight into the structural basis of the identified PRMT5‐pICln/RioK1/COPR5 interface was obtained from a co‐crystal structure of the TIM barrel domain in complex with peptide **23**, using data to a resolution of 2.55 Å (PDB ID: 7BOC).

The protein structure shows an unusual seven‐stranded β‐sheet with one antiparallel strand for the TIM barrel which is not observed in other PRMT5–MEP50 complexes.[^3a^, ^3c^] The conformation was confirmed by anomalous density maps using both the sulphur anomalous signal of Cys and Met as well as a platinum anomalous signal after soaking of the crystals in potassium tetrachloroplatinate solution (Figure S11). The reasons for the difference in the core structure are presumably the missing MEP50 interaction partner as well as a shortening of the PRMT5 construct. The region spanning residues 1–40 and 52–76 normally interacting and wrapping around the MEP50 insertion finger as well as region 165–178, constituting a loop interfacing with MEP50, were unstructured in this case. In order to gain a better understanding of the observed structure, TIM barrel residues significantly contributing to the PPI with MEP50 were predicted using the DrugScore^PPI^ web‐service, where a virtual alanine scan with a measurement of the change in the binding free energy (ΔΔ*G*) of the protein–protein complex (PDB ID: 4GQB) was performed (Table S6).^[14]^ As expected, the majority of the identified residues with high ΔΔ*G* values (>1 kcal/mol), and thus, high contribution to the PPI, were present in the disordered protein fragments (Arg62, Arg68 as well as Arg164, Asp165, Ile167, Ile168 and Asn170), supporting the observation of the MEP50 stabilisation effect on the TIM barrel.

**Figure 2:**
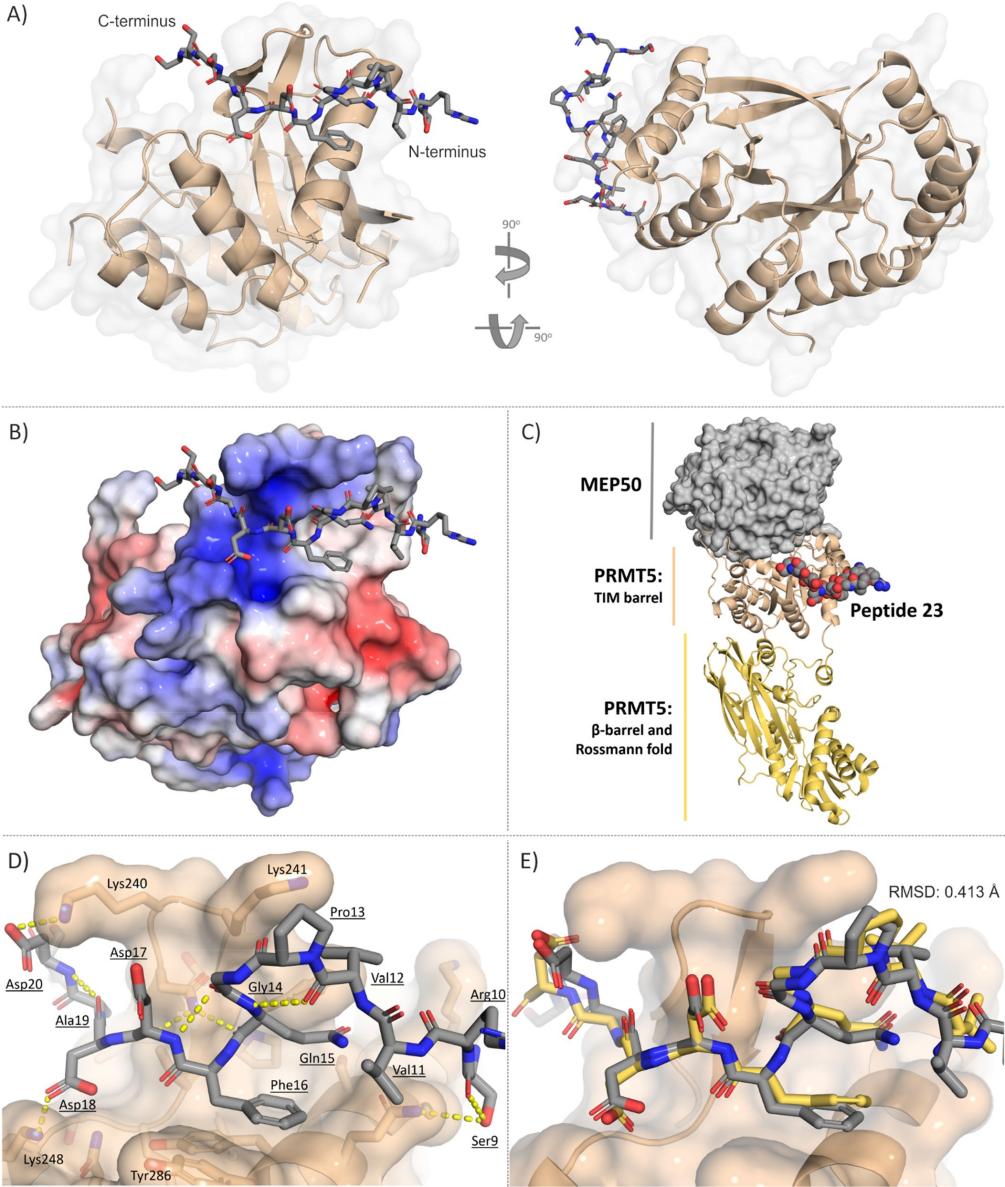
Crystallographic elucidation of the PRMT5–RioK1 interaction. A) Structure of the isolated TIM barrel of PRMT5, co‐crystallised with the RioK1‐derived peptide **23** (PDB ID: 7BOC). B) Structure of the TIM barrel–peptide complex (PDB ID: 7BOC). The colours of the protein surface show the electrostatic potential (blue=positive, red=negative, white=neutral). C) Structure of **23** fitted onto the full PRMT5–MEP50 complex. Image generated through a superposition of the obtained TIM barrel–peptide crystal structure with the PDB structure 4GQB. D) Close‐up of **23** bound to the TIM barrel. E) Superposition of the RioK1‐derived peptides from PDB structures 7BOC (grey, this work) and 6V0N (yellow, Sellers et al.).^[7]^

The peptide is wedged into a shallow groove of the TIM barrel, formed between Lys240/241, Lys248 and Tyr286 (Figure 2A, B and D). A significant part of the groove is positively charged and seems to interact electrostatically with the negatively charged end of the peptide, whereas the remaining area of the binding site is hydrophobic (Figure 2B). The C terminus of the peptide (Ser21/22) is fixed by the crystal symmetry molecule, while the N‐terminal part (Ser9, Arg10 and Val11) is strongly solvent exposed. The core of the sequence (GQFDDAD), however, is forming significant interactions with the protein groove. The peptide side chain of Asp20 interacts with Lys240, although, judging based on the alanine scan results (compound **27**, Table 3), the interaction is weak. Asp18 is in position to form a salt bride with Lys248. Interestingly, the side chain of Asp17 does not appear to create any salt bridge or hydrogen bonds with the protein and is solvent exposed. Phe16 interacts with the underlying α‐helix of the protein (Tyr283/286) through hydrophobic interactions, and as determined in the alanine scan, this interaction contributes significantly to the binding affinity of the peptide. Moreover, the backbone amide bond oxygens of Gln15 and Asp17 connect to PRMT5 side chain of Asn239 through hydrogen bonds. The peptide orientation in the groove and its conformation is further stabilised by a peculiar S‐shaped double β‐turn, spanning from Val12 to Asp17, fixing the hot‐spot residues in the correct position (Figure 2D). Involvement of the backbone of Val12 and Pro13 (or analogously Glu/Val and Tyr/Ala in case of COPR5 and pICln) in the formation of the peptidic β‐turn, explains the significant loss of the affinity upon truncation of these residues (compound **9**, Table 2).

The obtained crystal structure was found to be in agreement with the results by Sellers et al. which were deposited during the writing of this manuscript.^[8]^ Although the crystal structure reported by Sellers et al. (PDB ID: 6V0N) was measured with the full PRMT5–MEP50 complex the results matched our findings closely. The position and conformation of the RioK1 peptide was highly similar with an RMSD of 0.413 Å (PDB ID: 6V0N; Figure 2E).

**Figure 3:**
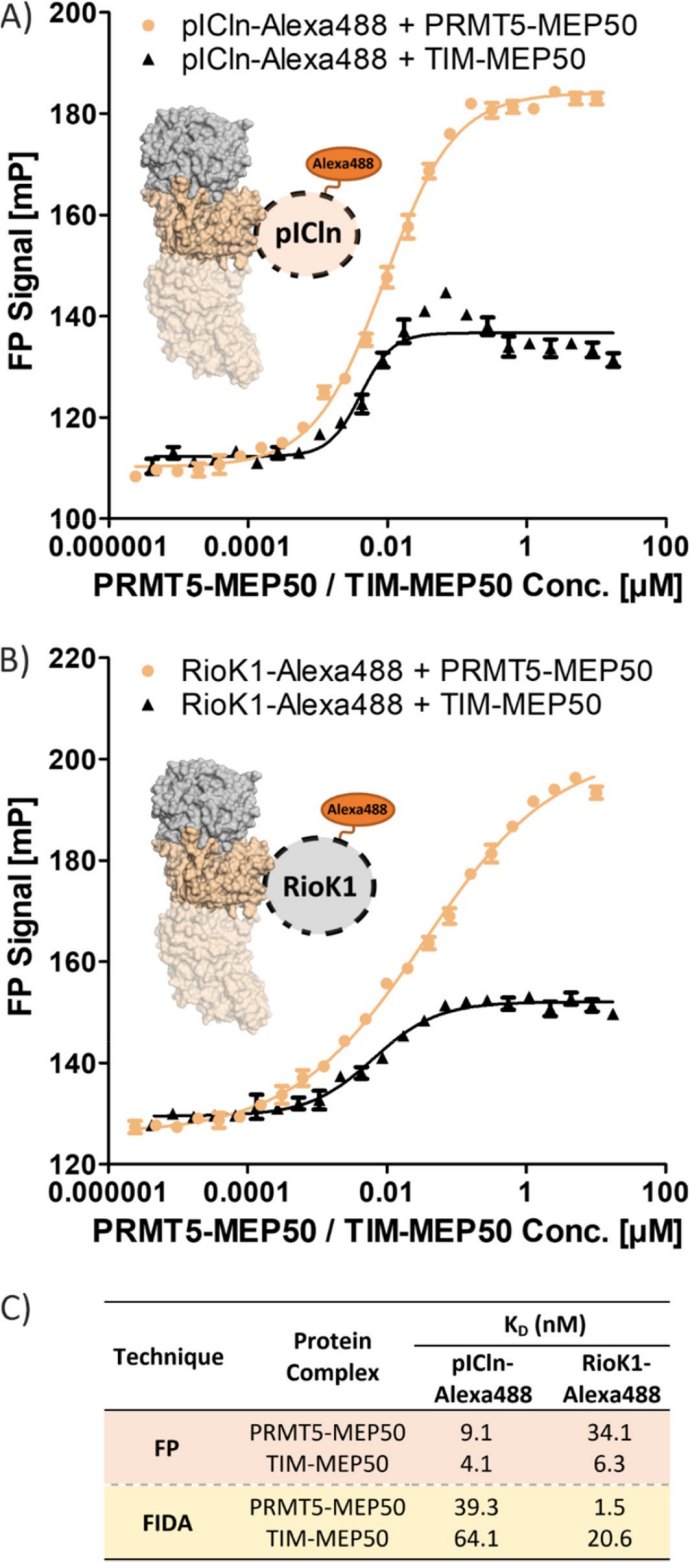
Interaction between pICln/RioK1 and PRMT5 protein complexes. A) FP results for the interaction of Alexa488‐labelled pICln with PRMT5–MEP50 and TIM–MEP50 (*n*=3). B) FP results for Alexa488‐tagged RioK1 interacting with PRMT5–MEP50 and TIM–MEP50 (*n*=3). C) Comparison of the *K*_D_ values obtained for the interactions between pICln/RioK1 and the PRMT5 protein complexes with FP and FIDA.

In order to further evaluate the interaction, the PRMT5–MEP50 complex and the full‐length adaptor proteins pICln and RioK1 we conducted FP experiments using labelled proteins. To this end, both pICln and RioK1 were labelled with the fluorescent dye Alexa488, and their binding to PRMT5–MEP50 and TIM–MEP50 was determined by means of FP measurements. The assay indicated that both proteins have high affinities for the PRMT5 constructs, where pICln afforded a *K*_D_ of 9.1±1.5 and 4.1±0.6 nM for PRMT5–MEP50 and TIM–MEP50, respectively (Figure 3A and C), and RioK1 gave a *K*_D_ of 34.1±9.9 nM for PRMT5–MEP50 and 6.3±0.2 nM for TIM–MEP50 (Figure 3B and C). These results were further supported by an assessment of the pICln/RioK1 interactions with the PRMT5 protein complexes using flow induced dispersion analysis (FIDA). FIDA allows for analyte‐indicator binding determination based on a detection of changes in diffusion rates of the analysed particles, where the diffusion is dependent on the fractions of the free and complexed indicator.^[15]^ The FIDA‐based experiments with pICln afforded a *K*_D_ of 39.3 nM for PRMT5–MEP50 and 64.1 nM for TIM–MEP50, and *K*_D_ of 1.5 and 20.6 nM for interactions between RioK1 and PRMT5–MEP50 and RioK1 and TIM–MEP50, respectively (Figures 3C and S7). In comparison to the peptides tested by fluorescence polarization, the full pICln and RioK1 proteins have a higher affinity. The higher affinity could indicate that other parts of the adaptor proteins are involved in the interaction. The report of 2009 by Pesiridis et al. implicated a PH domain of pICln as a second PRMT5 binding region, in addition to the AD3 acidic region containing the sequence of interest (GQFEDAD)^[4b]^ and it could be possible that a similar circumstance occurs for the binding of RioK1 to the methyltransferase. However, the obtained results can also reflect a preorganisation of the binding site in the context of the full‐length adaptor proteins.

## Summary and Conclusion

Herein, we have reported the biochemical determination of the protein–protein interaction interface on the TIM‐barrel domain of PRMT5, which binds the GQF[D/E]DA[E/D] sequence of pICln, RioK1, and COPR5. Our findings are in agreement with recently reported investigations by Sellers et al.^[7]^ Unique PRMT5 truncations containing an isolated TIM barrel domain or the TIM–MEP50 complex combined with the synthesis of a series of representative peptides derived from the PRMT5 adaptor proteins yielded further insight into the molecular and structural basis for these interactions. Through fluorescent labelling of pICln and RioK1, we were able to determine that the adaptor protein interactions are of high potency. However, suitable inhibitors might be able to disrupt the PPI as a novel therapeutic strategy, as was shown by the recent preliminary report of the group of Ianari.^[16]^

## Conflict of interest

H.A. was employed by AstraZeneca.

## Supporting information

As a service to our authors and readers, this journal provides supporting information supplied by the authors. Such materials are peer reviewed and may be re‐organized for online delivery, but are not copy‐edited or typeset. Technical support issues arising from supporting information (other than missing files) should be addressed to the authors.

Supplementary
