# Supplementary material for: Biochemical Investigation of the Interaction of pICln, RioK1 and COPR5 with the PRMT5–MEP50 Complex
Source: Chembiochem. 2021 Mar 31;22(11):1908–14. doi: 10.1002/cbic.202100079 (PMC8252068; doi:10.1002/cbic.202100079)
Supplement: Supplementary file 1 — Supplementary [file CBIC-22-1908-s001.pdf]

ChemBioChem

Supporting Information

Biochemical Investigation of the Interaction of pICln, RioK1 and COPR5 with the PRMT5–MEP50 Complex

Adrian Krzyzanowski, Raphael Gasper, Hélène Adihou, Peter 't Hart,* and Herbert Waldmann*

Table of Contents

General Methods 3
Linear Peptide Synthesis 4
Protein Expression 7
Protein Purification 7
Protein Labelling with Alexa 488 7
Fluorescence Polarisation (FP) 8
  Direct Binding Assay 8
  Competitive Binding Assay 8
FP Plots 9
Flow Induced Dispersion Analysis (FIDA) 12
Mass Photometry 13
MTase-Glo^{TM} Activity Assay 14
Crystallography – Protein Crystallisation, Data Collection and Analysis 15
DrugScore^{PPI} 20
HPLC Traces 21
References 34

General Methods

All chemical reagents and solvents, unless stated otherwise, were purchased from commercial suppliers: Sigma-Aldrich, Merck, Novabiochem, Acros Organics, abcr, Activate Scientific, Fluorochem, Carbolution Chemicals, Carbosynth, Iris Biotech, Carl Roth, TCI Deutschland, Fisher Scientific, Fisher Chemicals, Biosolve, Chem-Impex, Serva Electrophoresis, Gerbu Biotechnik, Thermo Scientific or VWR International. All air and moisture sensitive reactions were performed under an inert atmosphere of Ar gas. Organic solvents for moisture sensitive reactions were dried through storage over activated 3Å molecular sieves, for at least 24h. Automated peptide synthesis was performed using either CEM Microwave Peptide Synthesiser connected to CEM Discover microwave reactor (CEM Corporation, USA) or Syro I peptide synthesiser (Multisyntech GmbH, Germany). Preparative scale HPLC purification of the peptides was carried out either on an Agilent Infinity or Infinity II LC-MS system (Agilent Technologies, USA) equipped with a 125 mm x 21 mm, 5 µm or 125 mm x 10 mm, 5 µm Macherey-Nagel Nucleodur C18 Gravity column (Macherey-Nagel GmbH & Co. KG, Germany) and detection at 210 nm. Purity of the final products was determined at 210 nm using an Agilent Infinity HPLC system with 50 mm x 3 mm, 1.8 µm Macherey-Nagel Nucleodur C18 Gravity column, with a flow rate of 0.56 ml/min, and the samples obtained from the stability assay were tested using 125 mm x 3 mm, 5 µm Macherey-Nagel Nucleodur C4 Gravity at a flow rate of 1 ml/min, in both cases using elution system: 5% → 65% MeCN (0.1% TFA) in H_2O (0.1% TFA) over 14 min. HRMS analyses were performed on a LTQ Orbitrap mass spectrometer (Thermo Fisher Scientific, USA) using electrospray ionization. Concentrations of PRMT5-MEP50, TIM-MEP50 and TIM were measured with the Bradford assay, using Protein Assay Dye Reagent from Bio-Rad (Cat. #500-0006; Bio-Rad Laboratories, USA) and dilutions of BSA for calibration, whereas the concentrations of pICln and RioK1 were determined using NanoDrop 2000c Spectrophotometer (Thermo Fisher Scientific, USA). Fluorescence- and luminescence-based readouts were performed on the Spark Multimode Microplate Reader (Tecan Trading AG, Switzerland). Fluorescence-based assays were carried out in black, low volume, round bottom 384-well plates (ref. 4514, Corning Incorporated, USA), and the luminescence-based assays in white, low volume, round bottom 384-well plates (ref. 4512, Corning Incorporated). Mass photometry analyses were done on the OneMP mass photometer (Refeyn Ltd, UK). Flow induced dispersion analysis (FIDA) was performed using Fida 1 platform (FIDA Biosystems ApS, Denmark) with LED 488 nm detector and the analysis was done using FIDA software Version 2.01 (FIDA Biosystems ApS, Denmark).

Linear Peptide Synthesis

Linear peptides were synthesised on a polystyrene-based Rink Amide AM resin (substitution ca. 0.7 mmol/g, 100-200 mesh), or in case of the histone tail peptides on the polyethylene glycol-based Rink Amide ChemMatrix resin (substitution ca. 0.5 mmol/g), using standard Fmoc chemistry and solid-phase peptide synthesis (SPPS) methods.[1]

Peptide synthesis was performed using 4 equiv of amino acid, 4 equiv of PyBOP and 8 equiv of DIPEA in DMF. Fmoc removal was done with 20% piperidine solution in DMF containing 0.5M oxyma pure, in order to supress the aspartimide formation.[2]

The coupling of amino acids on the CEM synthesiser was carried out for 5 min at 75 ^{o}C under microwave irradiation (MWI), with an exception for arginines, where a modified coupling method was applied (rt to 75 ^{o}C, 30 min under MWI, followed by a second coupling at 75 ^{o}C over 5 min under MWI). Fmoc removal was performed at 75 ^{o}C under MWI for 30s followed by a second deprotection at 75 ^{o}C for 3 min. The peptide synthesis conducted on the Syro synthesiser was done through double 50 min amino acid coupling at rt. Fmoc removal was done twice at rt for 5 min for each residue.

Where applicable, acetylation of the resin-bound peptides was performed using Ac_2O (10 equiv) and DIPEA (12 equiv) in DMF over 30 min at rt.

In order to facilitate the C-terminal FITC labelling of the peptides (**7-10** and **20-22**), sidechains of the C-terminal lysines were protected by the acid sensitive Mtt group instead of the standard Boc group. Mtt was selectively removed by the treatment of the resin-bound peptides with 40% HFIP solution in DCM containing 2.5% TIPS, 4x 20 min at rt.

In all cases, Fmoc-AEEA-OH (4 equiv) was double-coupled to the peptide using the manual SPPS with PyBOP (4 equiv) and DIPEA (8 equiv) in DMF at rt over 2h, and Fmoc was cleaved with 20% piperidine in DMF with 0.5 M oxyma pure over 5 min, and then 10 min, at rt. The PEGylated peptide sequence was reacted with FITC isomer I (2 equiv) in presence of DIPEA (4 equiv) in DMF over 1h at rt, and the labelling with FITC was repeated overnight. Global deprotection and cleavage from the resin was achieved through treatment with TFA/H_2O/DODT/TIPS (90 : 5 : 2.5 : 2.5 v/v) over 2-3h at rt, or over 6h in the case of the histone tail peptides (**17-22**). The peptides were triturated in cold Et_2O and washed three more times with cold Et_2O, dried, dissolved in MeCN:H_2O (1:1 v/v; containing 0.1% TFA) and lyophilised. The crude peptides were purified by HPLC (using MeCN + 0.1% TFA and H_2O + 0.1% TFA as buffers) to afford the final products.

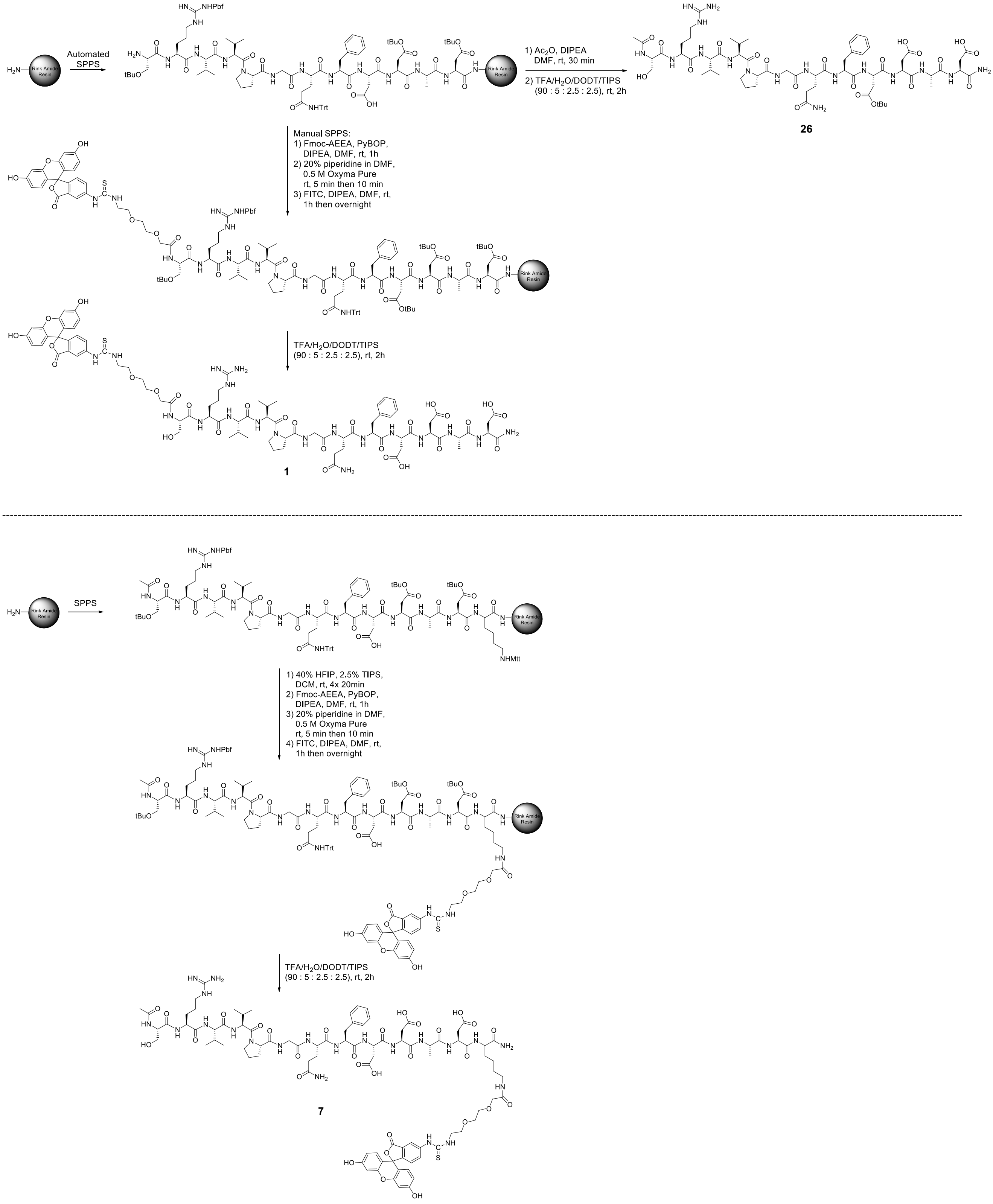

Scheme S1. Exemplary synthetic schemes leading to acetylated, N-terminally and C-terminally FITC-labelled linear peptides (compounds **26**, **1** and **7**).

Table S1. Sequences, K_D values and HRMS results for linear FITC labelled peptides.

Name	Sequence	K_D (nM)[a]	HRMS	
			m/z calculated	m/z found
**1**	Fitc-O2Oc-SRVVPGQFDDAD-NH2	522 ± 45	1838.73264 $[M+H]^+$	1838.74403
**2**	Fitc-O2Oc-TPTVAGQFEDAD-NH2	1145 ± 38	1783.67921 $[M+H]^+$	1783.68691
**3**	Fitc-O2Oc-MVFETGQFDDAED-NH2	549 ± 119	1018.86367 $[M+2H]^{2+}$	1018.86502
**4**	Fitc-O2Oc-PTVAGQF-NH2	>5000	1252.49794 $[M+H]^+$	1252.50244
**5**	Fitc-O2Oc-SRVVPGQFDD-NH2	>5000	1652.66858 $[M+H]^+$	1652.66956
**6**	Fitc-O2Oc-SRVVPGQF-NH2	>5000	1422.61470 $[M+H]^+$	1422.61635
**7**	Ac-SRVVPGQFDDADK(O2Oc-Fitc)-NH2	716 ± 36	1004.92272 $[M+2H]^{2+}$	1004.92569
**8**	Ac-VPGQFDDADK(O2Oc-Fitc)-NH2	208 ± 74	1666.63658 $[M+H]^+$	1666.63968
**9**	Ac-GQFDDADK(O2Oc-Fitc)-NH2	>5000	1470.51544 $[M+H]^+$	1470.51881
**10**	Ac-FDDADK(O2Oc-Fitc)-NH2	>5000	1285.43540 $[M+H]^+$	1285.43804
**11**	Fitc-O2Oc-SRVVPGQFDDADSSD-NH2	295 ± 11	1064.41546 $[M+2H]^{2+}$	1064.41816
**12**	Fitc-O2Oc-LLMSRVVPGQFDDAD-NH2	192 ± 26	1098.47426 $[M+2H]^{2+}$	1098.47733
**13**	Fitc-O2Oc-LLMSRVVPGQFDDADSSD-NH2	279 ± 41	1243.01976 $[M+2H]^{2+}$	1243.02345
**14**	Fitc-O2Oc-TPTVAGQF-NH2	>5000	1353.54562 $[M+H]^+$	1353.54723
**15**	Fitc-O2Oc-TPTVAGQFEDADVDH-NH2	520 ± 40	1067.92038 $[M+2H]^{2+}$	1067.92328
**16**	Fitc-O2Oc-VDTTPTVAGQFEDAD-NH2	2378 ± 817	1049.91476 $[M+2H]^{2+}$	1049.91657

[a]As determined with FP using the native PRMT5-MEP50 complex.

Table S2. Sequences and HRMS results for FITC-labelled histone tail peptides.

Name	Sequence	HRMS	
		m/z calculated	m/z found
**17**	Fitc-O2Oc-SGRGKQGGKARAKAKTRSSRA-NH2	1346.18587 $[M+2H]^{2+}$	1346.18590
**18**	Fitc-O2Oc-SGRGKGGKGLGKGGAKRHRKV-NH2	1312.69059 $[M+2H]^{2+}$	1312.69193
**19**	Fitc-O2Oc-ARTKQTARKSTGGKAPRKQLATKAARKSA-NH2	1200.98125 $[M+3H]^{3+}$	1200.98243
**20**	Ac-SGRGKQGGKARAKAKTRSSRA-O2Oc-K(Fitc)-NH2	954.49485 $[M+3H]^{3+}$	954.49568
**21**	Ac-SGRGKGGKGLGKGGAKRHRKV-O2Oc-K(Fitc)-NH2	932.16466 $[M+3H]^{3+}$	932.16531
**22**	Ac-ARTKQTARKSTGGKAPRKQLATKAARKSA-O2Oc-K(Fitc)-NH2	755.01277 $[M+5H]^{5+}$	755.01310

Table S3. Sequences and HRMS results for linear acetylated peptides

Name	Sequence	HRMS	
		m/z calculated	m/z found
**23**	Ac-SRVVPGQFDDADSSD-NH2	1635.72449 $[M+H]^+$	1635.72651
**24**	Ac-TPTVAGQFEDAD-NH2	1291.58009 $[M+H]^+$	1291.58152
**25**	Ac-MVFETGQFDDAED-NH2	1544.62096 $[M+H]^+$	1544.62257
**26**	Ac-SRVVPGQFDDAD-NH2	1346.63352 $[M+H]^+$	1346.63623
**27**	Ac-SRVVPGQFDDAA-NH2	1302.64369 $[M+H]^+$	1302.64621
**28**	Ac-SRVVPGQFDAAD-NH2	1302.64369 $[M+H]^+$	1302.64633
**29**	Ac-SRVVPGQFADAD-NH2	1302.64369 $[M+H]^+$	1302.63783
**30**	Ac-SRVVPGQADDAD-NH2	1270.60222 $[M+H]^+$	1270.60403
**31**	Ac-SRVVPGAFDDAD-NH2	1289.61206 $[M+H]^+$	1289.61450
**32**	Ac-SRVVPAQFDDAD-NH2	1360.64917 $[M+H]^+$	1360.64443
**33**	Ac-SRVVAGQFDDAD-NH2	1320.61787 $[M+H]^+$	1320.61953
**34**	Ac-SRVAPGQFDDAD-NH2	1318.60222 $[M+H]^+$	1318.60393
**35**	Ac-SRAVPGQFDDAD-NH2	1318.60222 $[M+H]^+$	1318.60389
**36**	Ac-SAVVPGQFDDAD-NH2	1261.56952 $[M+H]^+$	1261.57166
**37**	Ac-ARVVPGQFDDAD-NH2	1330.63860 $[M+H]^+$	1330.63818
**S1**	Ac-SGRGKGGKGLGKGGAKRHRKV-NH2	1066.64103 $[M+2H]^{2+}$	1066.64198

Protein Expression

All proteins were expressed at the Protein Chemistry Facility (PCF) based in the MPI Dortmund.

For expression of the proteins, all constructs were subcloned into pOPIN vectors.

Full-length human pICln, N-terminally tagged with 6His-TRX followed by the 3C cleavage site, and TIM barrel domain (1-292 aa) of human PRMT5, N-terminally tagged with 6His-SUMO, were expressed in BL21 CodonPlus (DE3) RIPL strain of *Escherichia coli*. Bacteria with the respective plasmids were cultured in Terrific Broth medium. Expression of TIM barrel was auto-induced, with incubation of the starter-culture (starting OD of ~0.05) at 35 °C for 2.5h, followed by an overnight incubation (20-24h) at 25 °C. Expression of pICln was induced at OD ~0.6-0.8 using 0.3 mM IPTG *and E. coli* was incubated at 20 °C for 20h. Bacteria were harvested by centrifugation and lysed.

Expression of full-length human RioK1 tagged with 6NHis-3C and co-expression of full-length human PRMT5 (N-terminal 6His-MBP with 3C) with MEP50 (N-terminal 6His-TRX with 3C), and of TIM barrel (1-292 aa) with MEP50 (both N-terminally tagged with His-TRX with 3C cleavage site) was done using the Flash Bac Expression System (Oxford Expression Technologies, UK) with HighFive insect cells in Gibco Sf-900 III SFM medium. The cells were harvested by centrifugation and lysed.

Protein Purification

All proteins were purified at the PCF (MPI Dortmund).

Protein purification was performed on HisTrap FF crude 5 ml Ni-based column, followed by the on-column treatment with 6His-tagged HRV-3C PreScission protease, or in case of the SUMO-tagged TIM barrel, with 6His-tagged SUMO protease. The cleaved proteins were then further purified using gel filtration chromatography with the HiLoad 26/60 Superdex 75 or 200 prep grade column. PRMT5-MEP50, TIM-MEP50 and TIM barrel were stored in a buffer containing 50 mM HEPES, 250 mM NaCl, 1 mM TCEP with the pH adjusted to 8.0, whereas, pICln was stored in a buffer of 20 mM phosphate, 100 mM NaCl, 5 mM TCEP and pH of 7.5, whereas RioK1 was stored in a buffer of 50 mM Tris, 200 mM NaCl, 10% glycerol, 1 mM TCEP at pH 8.0.

Protein Labelling with Alexa 488

To a protein solution (ca. 1 mg/ml) in 0.2 M bicarbonate buffer with 1 mM TCEP at pH 8.3, was added 10 mM Alexa 488 NHS-ester in DMSO (8 equiv), and the mixture was kept on ice, in darkness, overnight. The protein was washed eight times with buffer (50 mM HEPES, 250 mM NaCl, 1 mM TCEP, 8.0 pH) using an Amicon® Ultra-0.5 mL spin filter. Protein concentration was then determined using the NanoDrop spectrophotometer.

Fluorescence Polarisation (FP)

Direct Binding Assay

The assay was performed in a buffer of 50 mM HEPES, 250 mM NaCl, 1 mM TCEP, 0.01% (v/v) Tween 20, pH 8.0, in black, 384 well-plates, with a total volume of 10 µl per well. The analysed, FITC labelled peptides, or Alexa488-labelled pICln or RioK1, were tested at a final concentration of 1 nM, and the appropriate unlabelled protein was titrated as two-fold dilution series. The plates were incubated at 20-21 ^{o}C for 30 min in case of FITC-labelled peptides or 1-2h in case of the Alexa488-labelled protein, and analysed on a plate reader using 485 nm excitation and 535 nm emission wavelength. The assay was performed in triplicates.

Competitive Binding Assay

The competitive binding assay was performed in a buffer of 50 mM HEPES, 250 mM NaCl, 1 mM TCEP, 0.01% (v/v) Tween 20, 8.0 pH, in black, 384 well-plates, with a total volume of 10 µl per well. The FITC labelled peptides were added at the final concentration of 1 nM, were mixed with PRMT5-MEP50 (at the final concentration of 600 nM), and the appropriate non-labelled peptide was titrated as two-fold dilution series. The plates were incubated at 20-21 ^{o}C for 30 min and analysed on a plate reader using 485 nm excitation and 535 nm emission wavelength. The experiment was performed in triplicates.

FP Plots

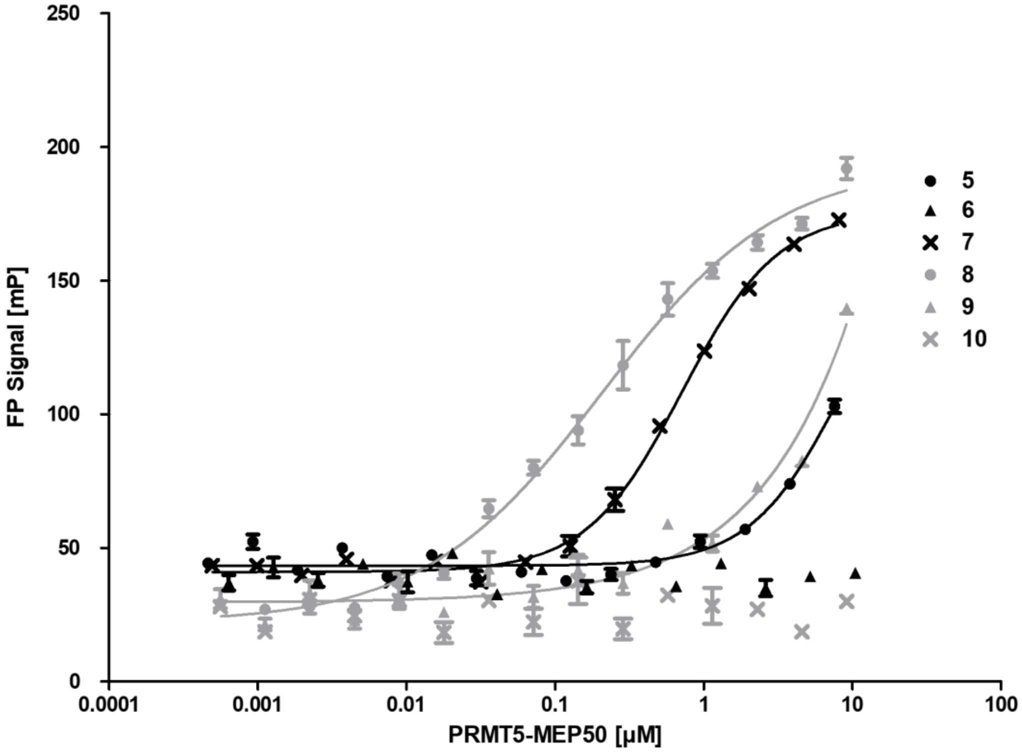

Figure S1. FP assay with PRMT5-MEP50.

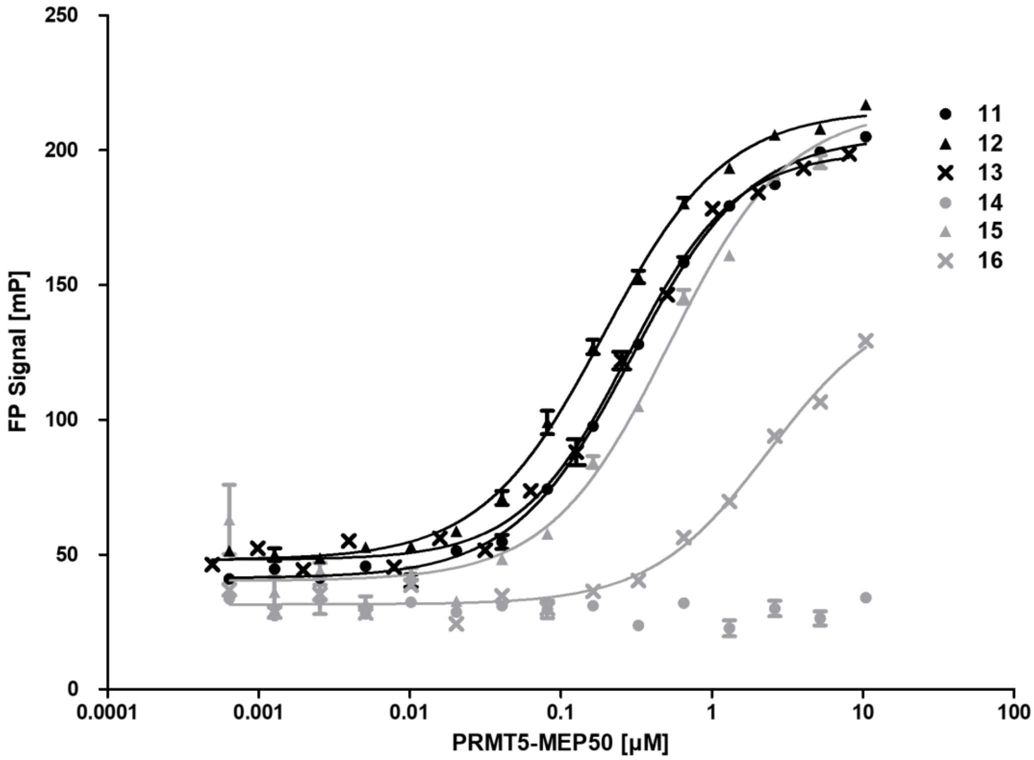

Figure S2. FP assay with PRMT5-MEP50.

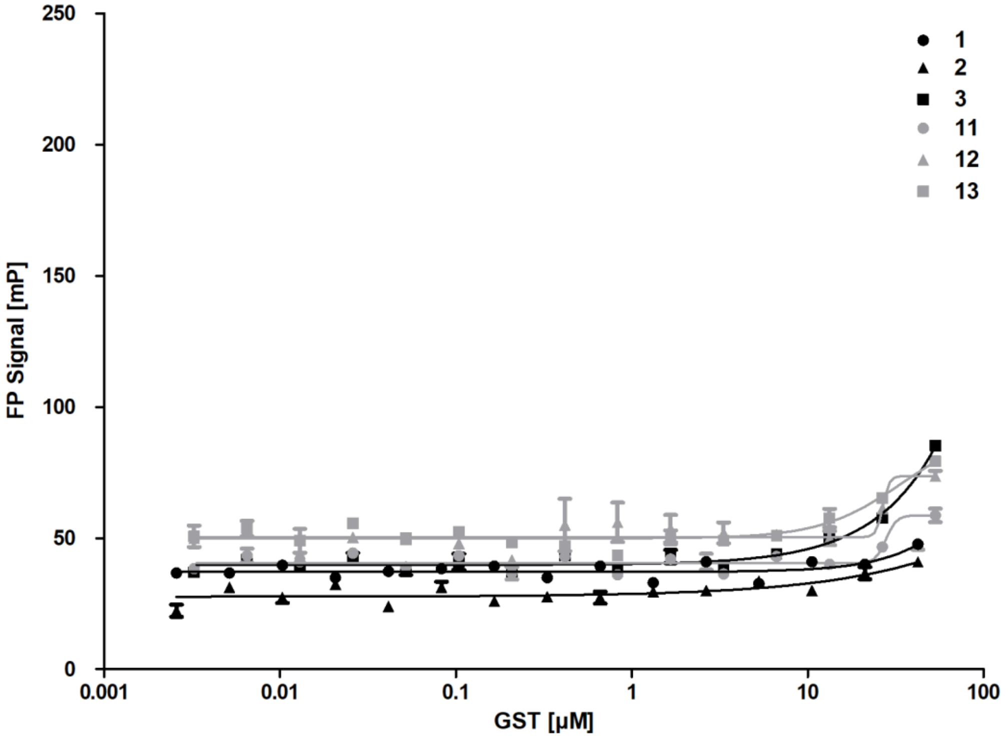

Figure S3. FP assay with GST.

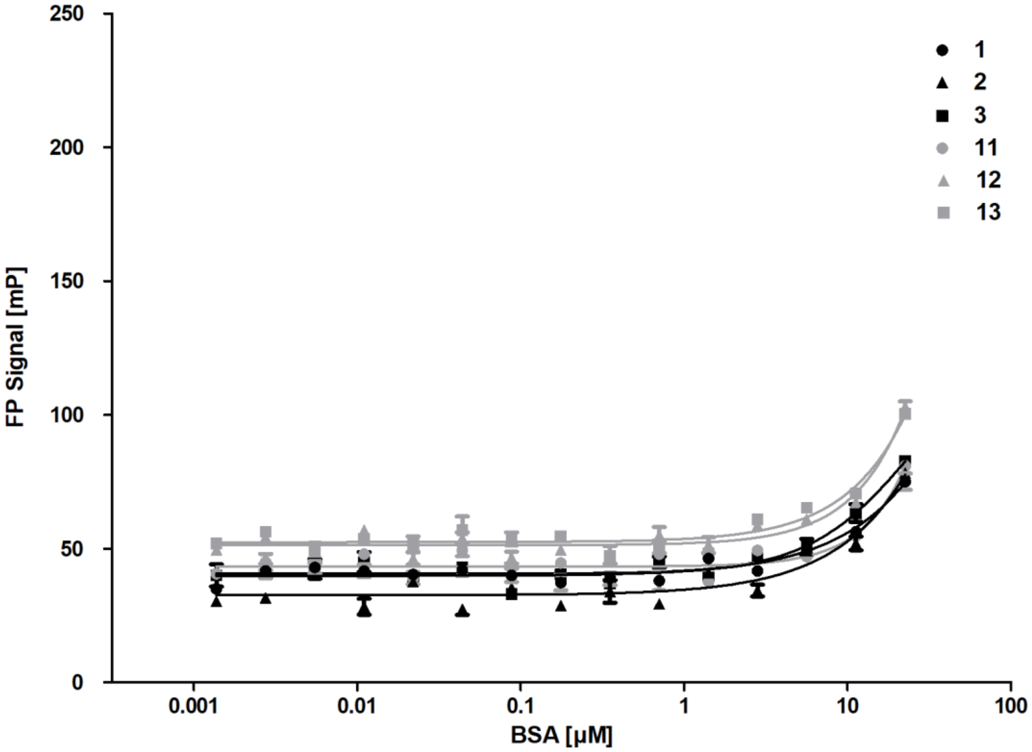

Figure S4. FP assay with BSA.

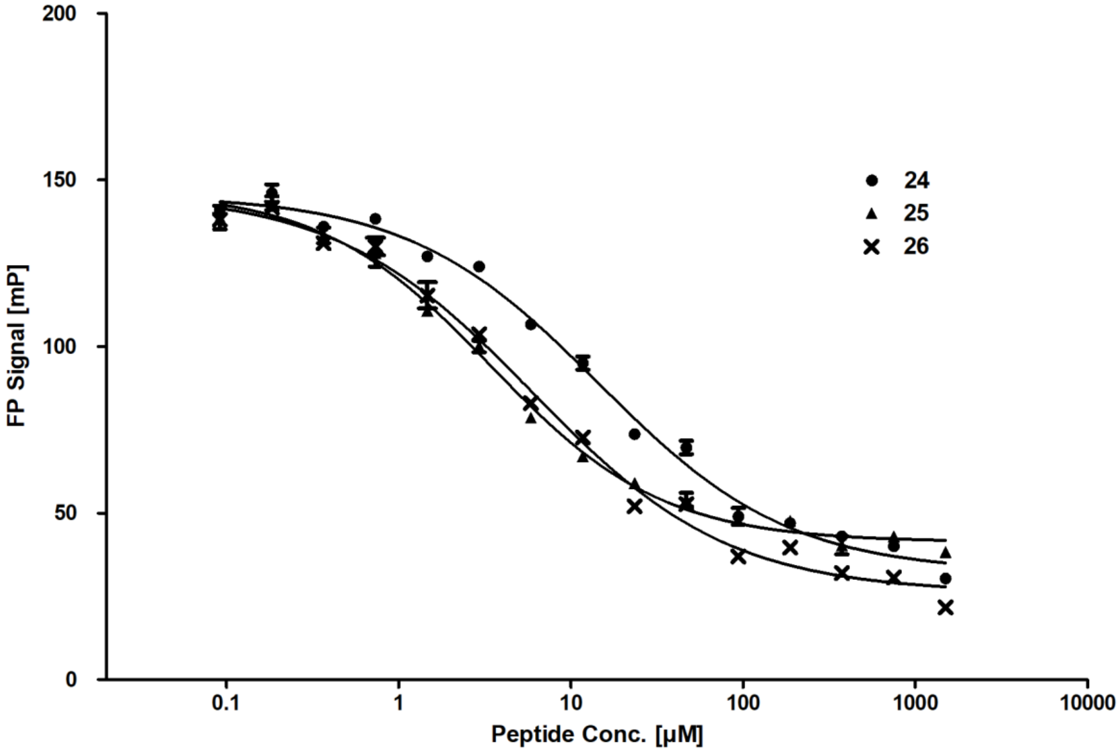

Figure S5. Competitive FP with PRMT5-MEP50 and peptide **11** used as a fluorescent tracer.

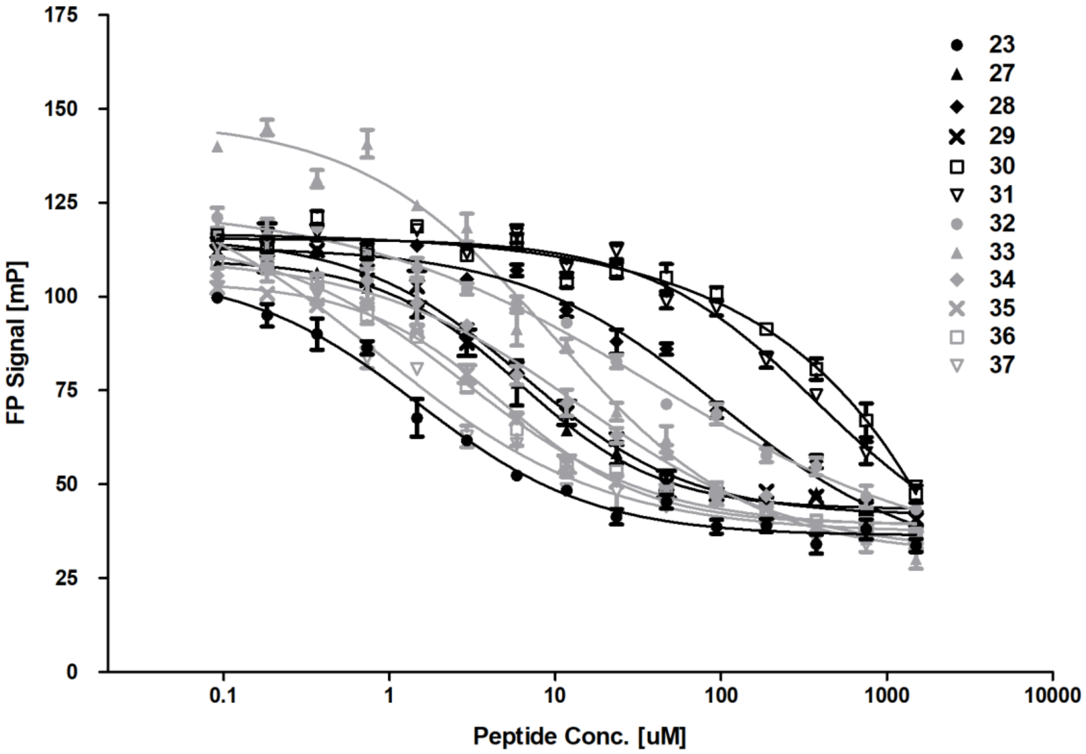

Figure S6. Competitive FP with PRMT5-MEP50 and peptide **11** used as a fluorescent tracer.

Flow Induced Dispersion Analysis (FIDA)

FIDA experiments were performed using Fida 1 platform (Biosystems ApS, Denmark) with LED 488 nm detector and PEG-coated capillary with inner diameter of 75 µm. The analyses were performed in a buffer of 50 mM HEPES, 250 mM NaCl, 1 mM TCEP, 8.0 pH and 0.01% (v/v) Tween 20. The column was equilibrated with buffer, followed by the injection of the indicator sample (Alexa488 labelled pICln or RioK1), and subsequent injection of the indicator-analyte mixtures (pICln-Alexa488 or RioK1-Alexa488 mixed with unlabelled PRMT5-MEP50 or TIM-MEP50). The indicator was used at a concentration of 10 nM. The analysis was performed at 25 °C. The obtained data were evaluated using FIDA software Version 2.01 (FIDA Biosystems ApS, Denmark) using fraction setting fixed to 75% or 100% and fixed selection of Rh for one species option. The samples were analysed as technical triplicate.

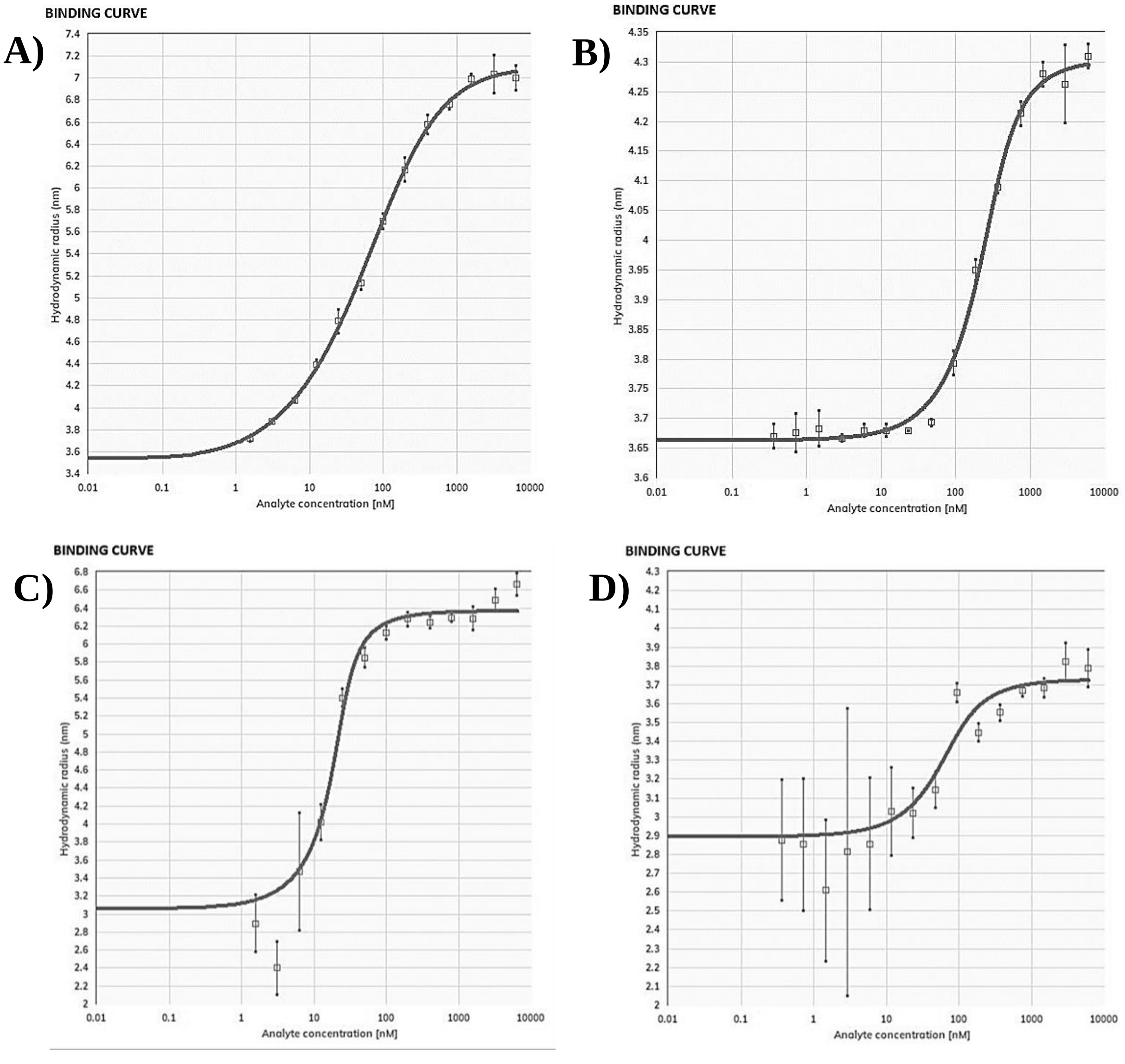

Figure S7. Binding curves for FIDA measurements. **A)** pICln-Alexa488 and PRMT5-MEP50. **B)** pICln-Alexa488 and TIM-MEP50. **C)** RioK1-Alexa488 and PRMT5-MEP50. **D)** RioK1-Alexa488 and TIM-MEP50.

Table S4. FIDA results for pICln and RioK1 interactions with PRMT5 protein complexes.

	pICln-Alexa488		RioK1-Alexa488	
	PRMT5-MEP50	TIM-MEP50	PRMT5-MEP50	TIM-MEP50
**Affinity constant**	39.26 nM	64.11 nM	1.53 nM	20.62 nM
**Indicator size**	3.53 nm	3.66 nm	3.06 nm	2.89 nm
**Complex size**	7.10 nm	4.30 nm	6.37 nm	3.73 nm
**R^2**	0.999	0.996	0.922	0.801

Mass Photometry

Autofocus of the mass photometer was set using 18 µl of the stock buffer of 50 mM HEPES, 250 mM NaCl, 1 mM TCEP, 8.0 pH at rt. To that was added 2 µl of either 500 nM PRMT5-MEP50 complex or 500 nM TIM-MEP50 complex solution in the stock buffer at rt, giving the final working concentration of 50 nM, followed by the acquisition of the movies and the automated data analysis.[3,4]

A)

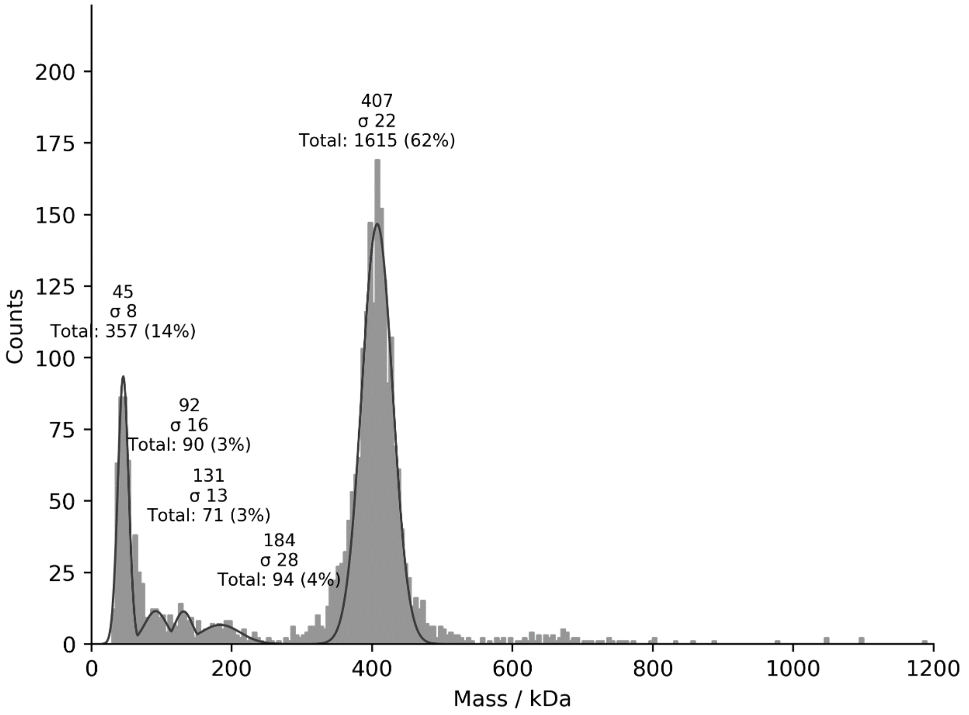

B)

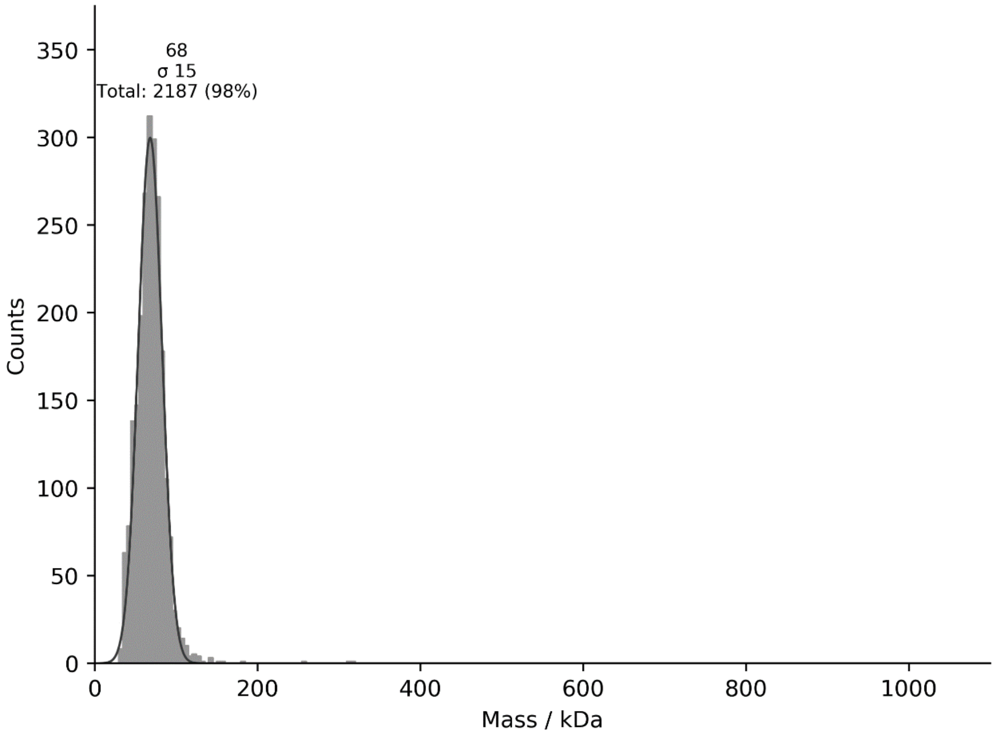

Figure S8. Mass distribution for the purified PRMT5-MEP50 complex (**A**, 407±22 kDa, heterooctamer) and TIM-MEP50 (**B**, 68±15 kDa, dimer), determined by mass photometry.

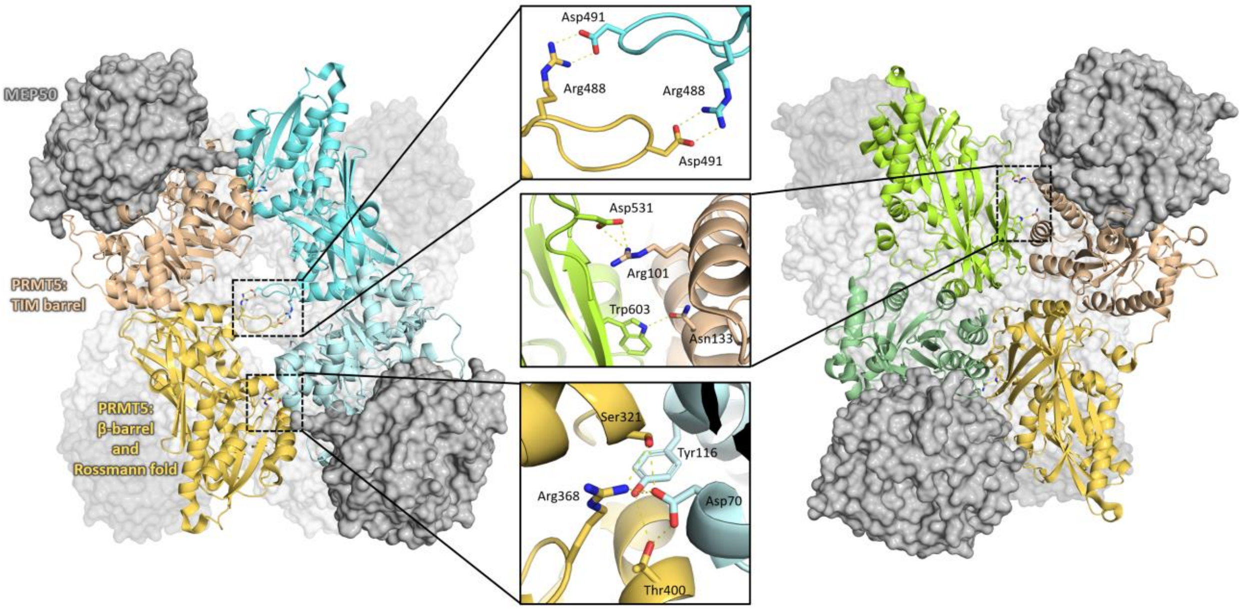

Figure S9. Depiction of the key PRMT5 interactions contributing to the hetero-tetramer ($PRMT5_2$-$MEP50_2$) and the hetero-octamer ($PRMT5_4$-$MEP50_4$) formation based on the 4GQB PDB structure.[5] PRMT5-MEP50 hetero-octamer is shown at two angles, with the second structure presented after 90° rotation.

MTase-Glo^{TM} Activity Assay

The methyltransferase activity of the expressed PRMT5-MEP50 complex was confirmed using the MTase-Glo^{TM} Methyltransferase Assay from Promega (Promega Corporation, USA).[6]

The assay was performed in a 1X reaction buffer of 20 mM Tris, 50 mM NaCl, 1 mM EDTA, 3 mM $MgCl_2$, 0.1 mg/ml BSA, 1 mM DTT at pH = 8.0, in white, 384 well-plates. A dilution series of the EPZ015666 PRMT5 inhibitor was prepared in a mixture of 2 µM SAM, 1 µM H4 histone peptide (**S1**) and 2X MTase-Glo^{TM} Reagent in the 1X reaction buffer (2.5 µl per well), and equal volume of 200 nM PRMT5-MEP50 protein solution was added to give a total volume of 5 µl per well, and incubated at 20-21 °C for 1h. MTase-Glo^{TM} Detection Solution (5 µl) was added, the plate was incubated at 20-21 °C for 1h, followed by a luminescence measurement at a plate reader. The experiment was performed as triplicates.

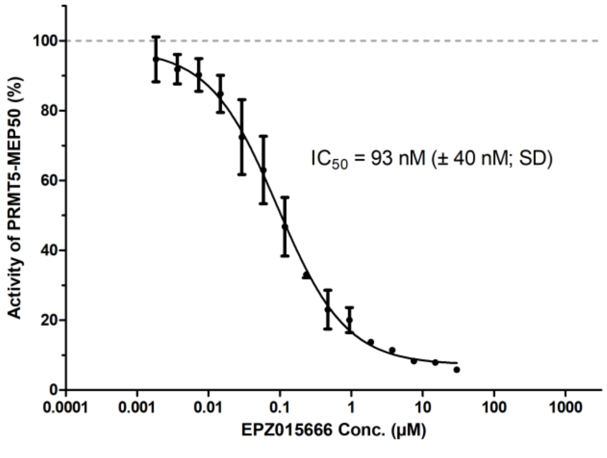

Figure S10. MTase-Glo^{TM} Methyltransfarase Assay performed on the expressed PRMT-MEP50 complex with the reference active-site methyltransferase inhibitor EPZ015666, confirming the expected enzymatic activity of the obtained protein complex.

Crystallography – Protein Crystallisation, Data Collection and Analysis

The protein was crystallised in a sitting drop setup, using 100 nl reservoir solution (0.1M NaAc, 0.2 M CaAc, 26-30% (v/v) PEG 400, at pH 4.18-4.73) and 100 nl of protein-peptide solution (5 mg/ml protein, 450 µM peptide **23**) in a buffer of 50 mM HEPES, 150 mM NaCl, 1 mM TCEP, 10% (v/v) glycerol and pH = 8.0. The crystals were grown for two weeks at 4 °C, and were flash frozen in liquid nitrogen. Datasets of the crystals were taken at the Suisse Light Source X10SA beamline (Table S6). The data were processed and scaled using Xia2/DIALS.[7,8] The structure could be solved using a truncated model of pdb 4X60 (chain A) with Phaser.[9] Cycles of manual and automated refinement was performed with phenix.refine and Coot.[10,11]

In order to produce anomalous data for structure verification we soaked crystals of TIM barrel-peptide complex in 2 mM solution of K_2PtCl_4 for 9 min. A dataset was taken at the X10SA beamline at a wavelength of 0.85Å. Similarly, another crystal of the complex was measured at X10SA at a wavelength of 2Å for sulphur anomalous data. Both datasets were solved using the previously refined TIM barrel-peptide structure as phaser search model and refined using phenix-refined. Anomalous maps were calculated from the mtz files in coot using ANOM and ANOMPH amplitudes and phases.

The Matthews coefficient of 4.6 deviates substantially from the ideal value of 2.3 indicating a solvent content of 73.25%. Attempts to place a second TIM barrel structure or parts thereof into the asymmetric unit have failed in every program tested. The MR rotation function score indicates the presence of a single TIM barrel molecule only in the asymmetric unit.

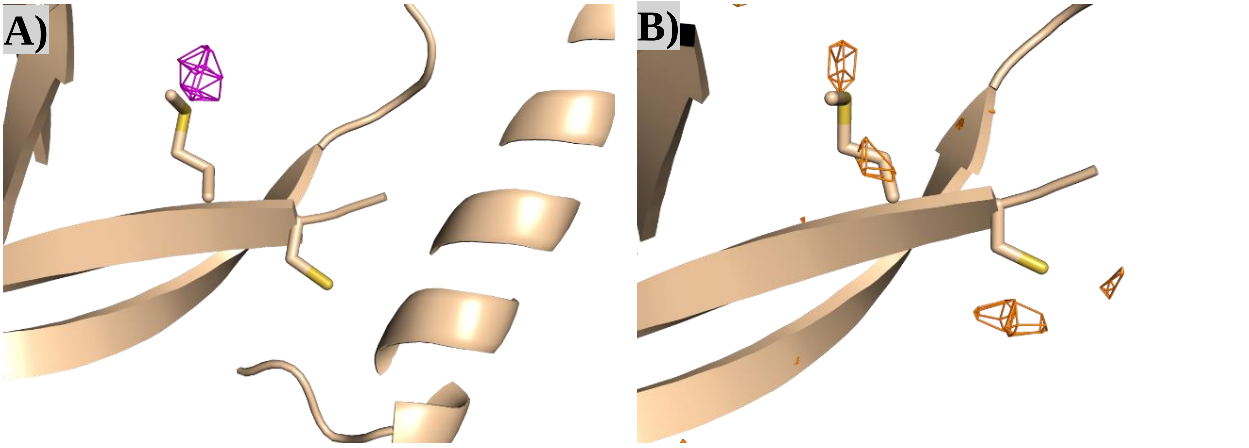

Figure S11. Anomalous density calculations. **A)** Crystal structure of TIM barrel domain showing the antiparallel strand with prominent residues Cys42 and Met43. The purple density represents the anomalous density of a Pt-derivative at 0.86Å at a contour level of 3.7. **B)** Crystal structure of TIM barrel domain zooming into the antiparallel strand. The orange density represents the anomalous density of a native crystal at 2.0Å at a contour level of 2.7.

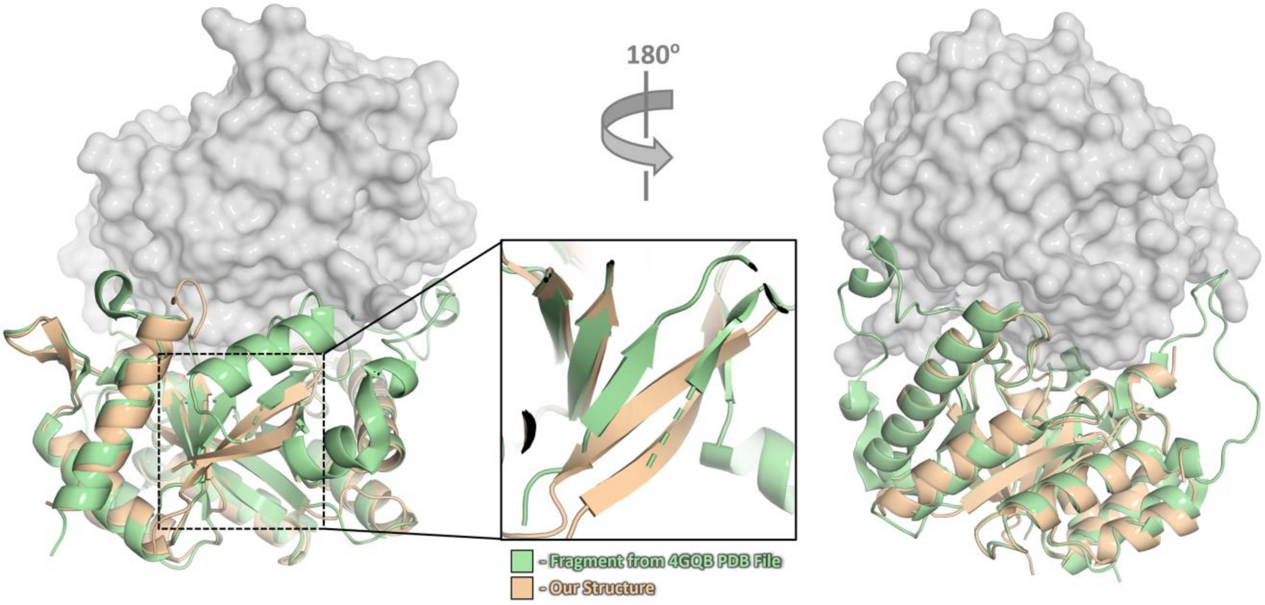

Figure S12. Crystallised TIM barrel (7BOC) superimposed onto a fragment of PRMT5-MEP50 (4GQB), with a close-up of the modified β-strand protein core, showing the discussed antiparallel strand.

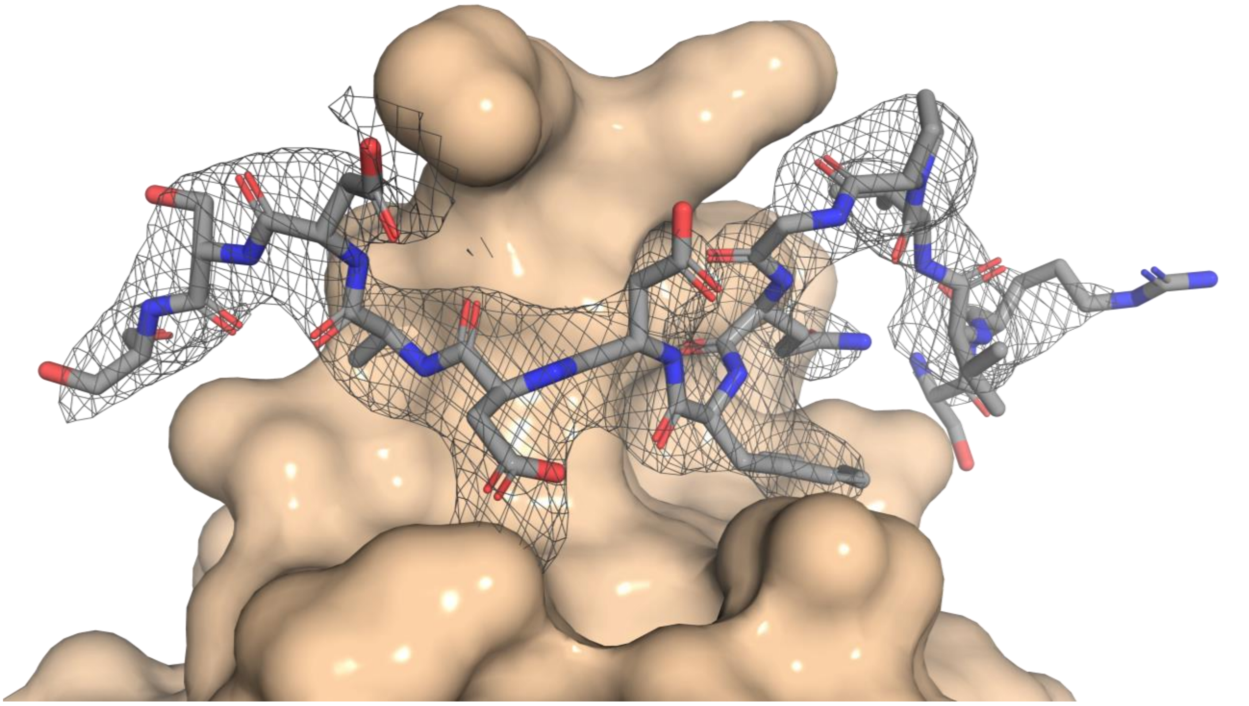

Figure S13. Peptide **23** bound to TIM barrel. The 2Fo-Fc electron density for the peptide is contoured at 2.5σ (PDB 7BOC).

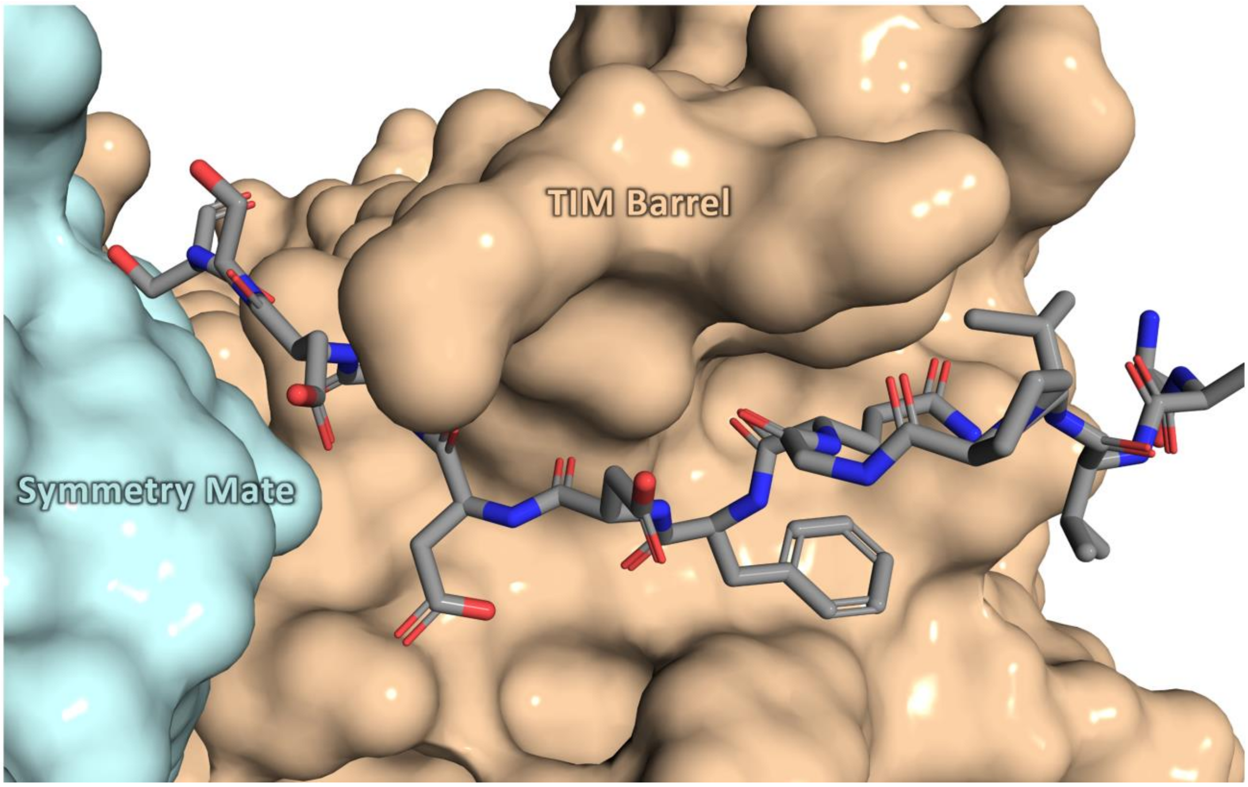

Figure S14. Visualisation of the TIM barrel symmetry partner. C-terminal fragment of **23** is fixed by the symmetry partner (PDB 7BOC).

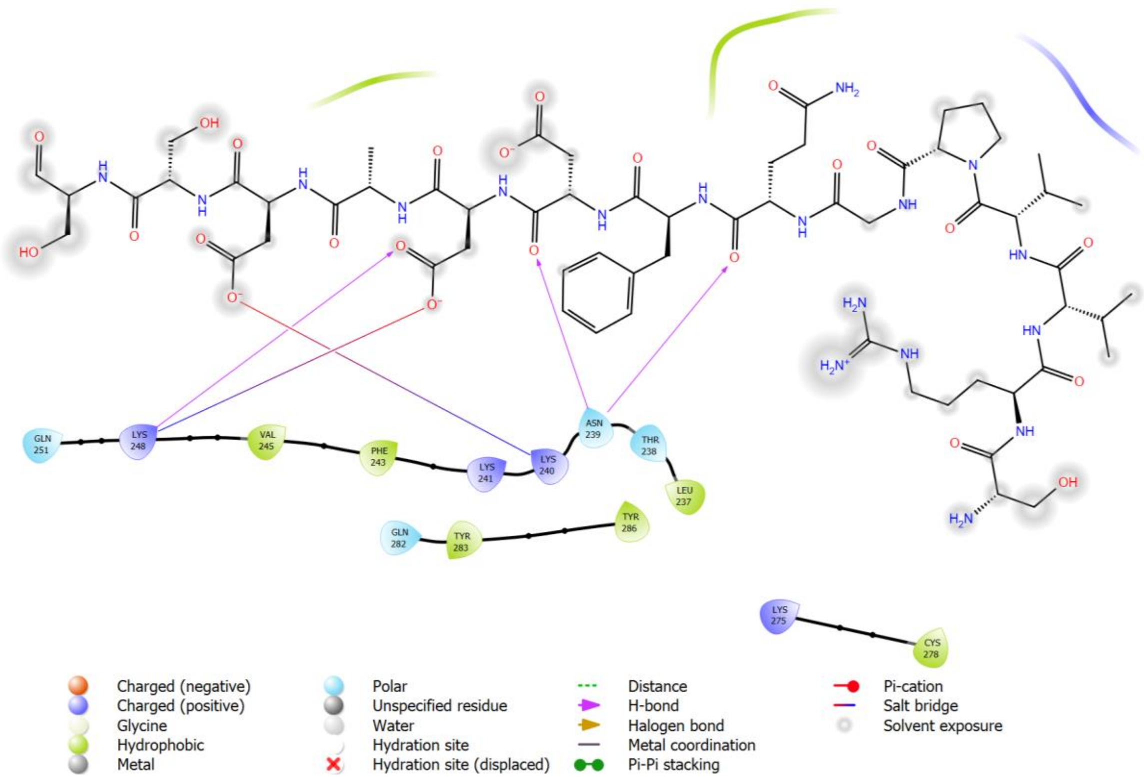

Figure S15. Ligand interaction diagram automatically generated for the TIM-peptide crystal structure using Maestro version 12.3.013, release 2020-1 (Schrödinger).

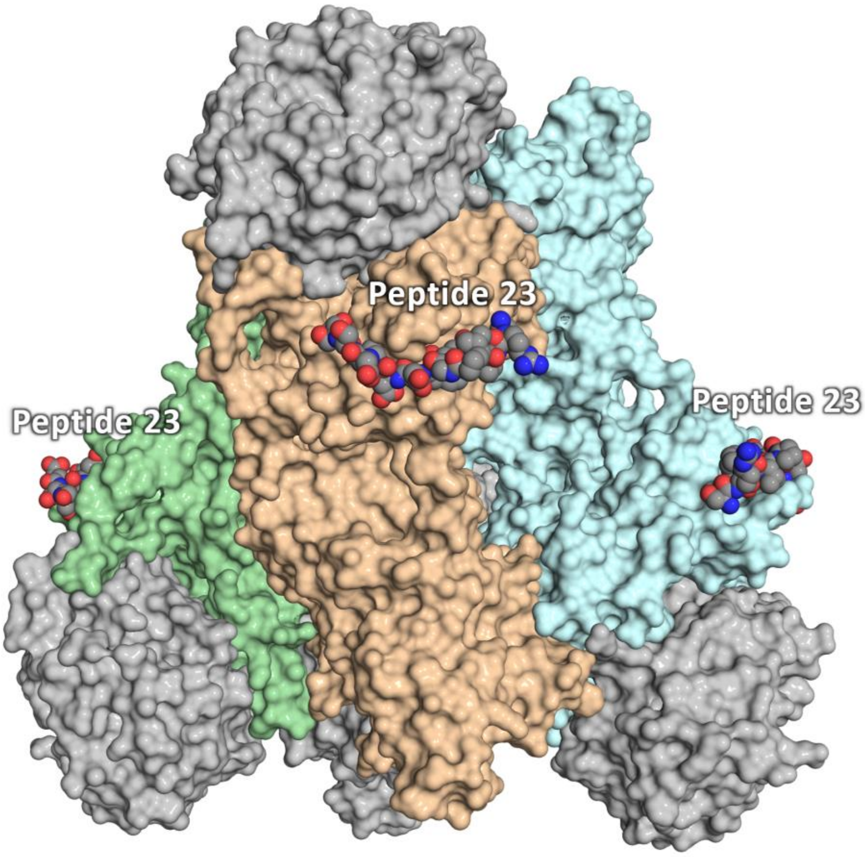

Figure S16. Crystal structure of **23** fitted onto hetero-octameric PRMT5-MEP50 complex. Image generated through a superposition of the obtained TIM-peptide crystal structure with the 4GQB PDB structure.

Table S5. Data collection and refinement statistics. Statistics for the highest-resolution shell are shown in parentheses.

**Wavelength**	0.99986
**Resolution range**	46.74 - 2.55 (2.641 - 2.55)
**Space group**	P 31 2 1
**Unit cell**	96.63 96.63 112.68 90 90 120
**Total reflections**	409259 (41998)
**Unique reflections**	20296 (1991)
**Multiplicity**	20.2 (21.1)
**Completeness (%)**	99.58 (99.50)
**Mean I/sigma(I)**	12.05 (1.33)
**Wilson B-factor**	71.65
**R-merge**	0.1916 (4.157)
**R-meas**	0.1966 (4.26)
**R-pim**	0.04392 (0.9258)
**CC1/2**	0.999 (0.829)
**CC***	1 (0.952)
**Reflections used in refinement**	20232 (1983)
**Reflections used for R-free**	1012 (99)
**R-work**	0.2269 (0.3443)
**R-free**	0.2552 (0.3418)
**CC(work)**	0.959 (0.819)
**CC(free)**	0.836 (0.751)
**Number of non-hydrogen atoms**	1844
**macromolecules**	1844
**Protein residues**	225
**RMS(bonds)**	0.010
**RMS(angles)**	1.30
**Ramachandran favored (%)**	94.01
**Ramachandran allowed (%)**	5.53
**Ramachandran outliers (%)**	0.46
**Average B-factor**	88.41
**macromolecules**	88.41
**Number of TLS groups**	7

DrugScore^{PPI}

Table S6. Results of the DrugScore^{PPI} analysis of the interface between the TIM barrel and MEP50 in 4GQB.[17]

**TIM Barrel Residue**	**$\Delta\Delta G_{calc}$ [kcal/mol]**	**Degree of Buridness**	**Possible Saltbridges**
Asn21A	0.54	5.89	No
Cys22A	0.31	5.29	No
Val23A	0.18	5.44	No
Glu25A	0.32	6.86	Yes
Ile26A	0.07	2.42	No
Met43A	0.11	3.67	No
Val45A	0.01	1.11	No
Phe46A	0.17	5.92	No
His47A	0.36	8.70	No
Arg49A	0.88	6.10	Yes
**Phe50A**	**1.01**	6.74	No
Lys51A	0.67	2.50	No
Arg52A	0.21	5.31	No
Glu53A	0.35	9.48	No
Gln56A	0.06	1.99	No
Glu57A	0.06	2.15	No
**Arg62A**	**1.04**	4.52	Yes
Gln66A	0.02	3.93	No
Thr67A	0.18	4.82	No
**Arg68A**	**3.22**	7.68	No
Ser69A	0.14	4.72	No
Leu72A	0.61	3.72	No
Lys85A	0.24	8.32	No
Trp89A	0.18	6.30	No
Arg91A	0.24	4.56	No
Ser94A	0.21	6.23	No
Lys95A	0.05	0.87	No
**Val96A**	**1.62**	6.43	No
Lys98A	0.47	1.68	No
Ile99A	0.64	4.50	No
Asn102A	0.29	7.10	No
Asn127A	0.14	4.81	No
Glu161A	0.64	5.37	Yes
Leu163A	0.31	4.50	No
**Arg164A**	**1.36**	6.80	No
**Asp165A**	**2.81**	3.78	No
Asp166A	0.37	5.07	No
**Ile167A**	**2.44**	3.67	No
**Ile168A**	**2.98**	8.28	No
Glu169A	0.01	3.10	No
**Asn170A**	**1.59**	5.89	No
Thr173A	0.21	7.33	No
His175A	0.11	3.80	No
Thr233A	0.27	8.52	No
Ser234A	0.06	1.60	No
Thr267A	0.21	7.33	No
The269A	0.63	6.08	No
Asn270A	0.17	8.32	No
His271A	0.31	7.59	No

HPLC Traces

Peptide 1

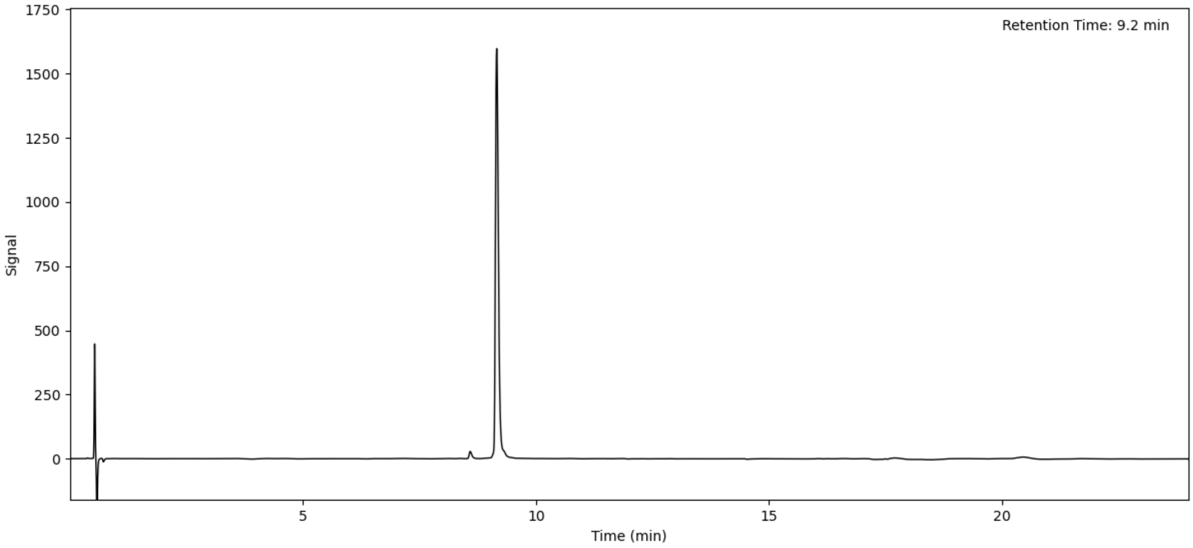

Peptide 2

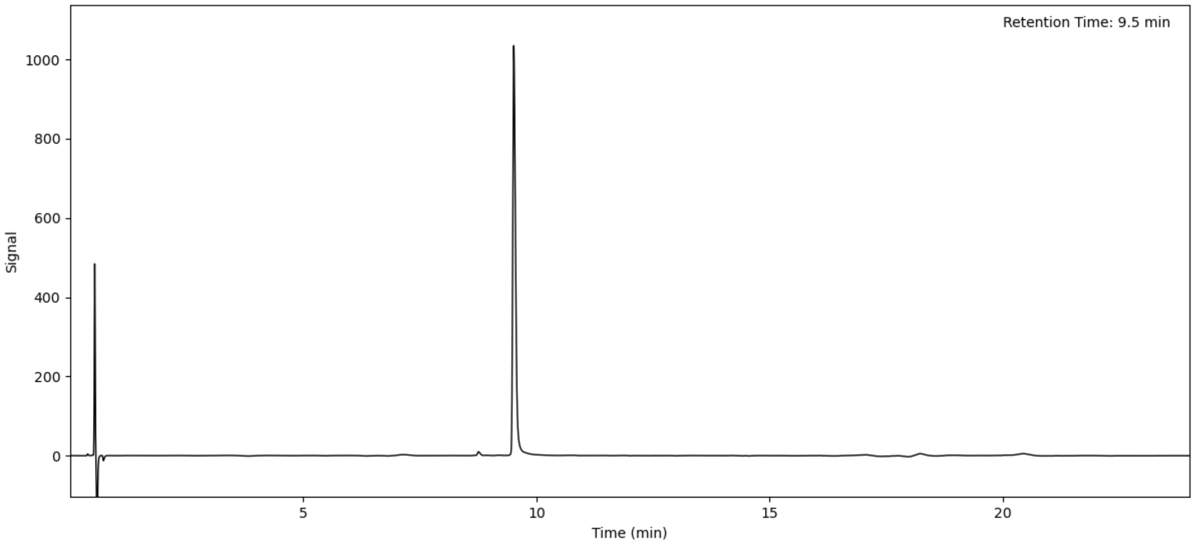

Peptide 3

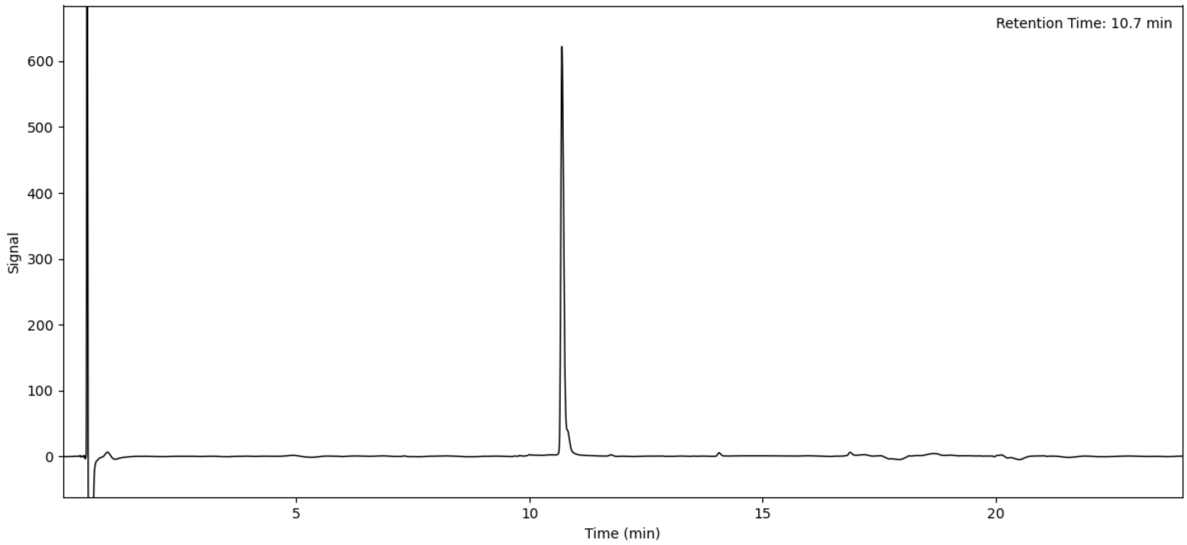

Peptide 4

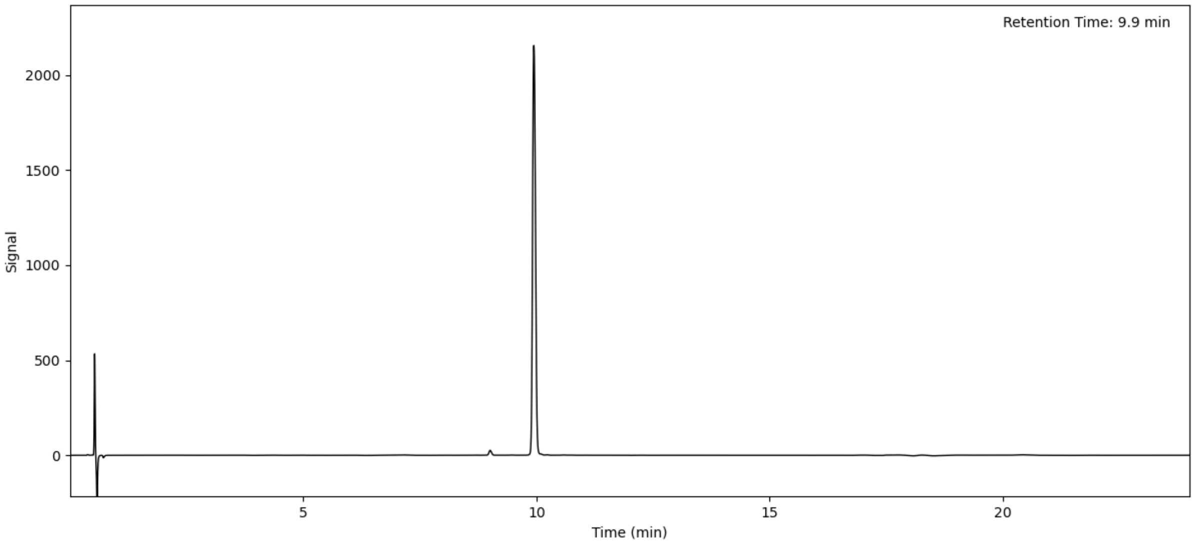

Peptide 5

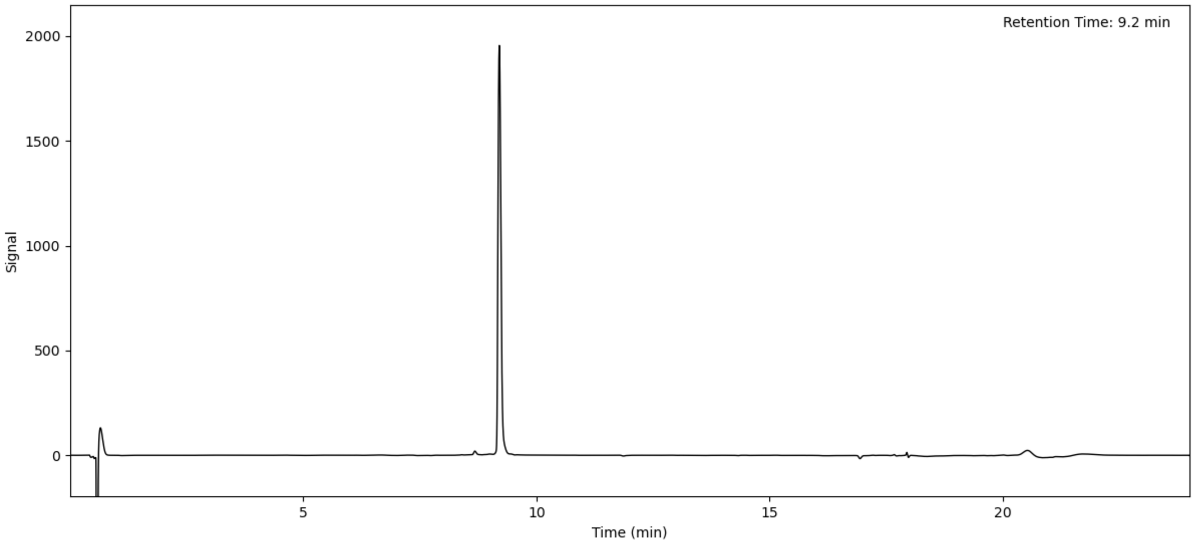

Peptide 6

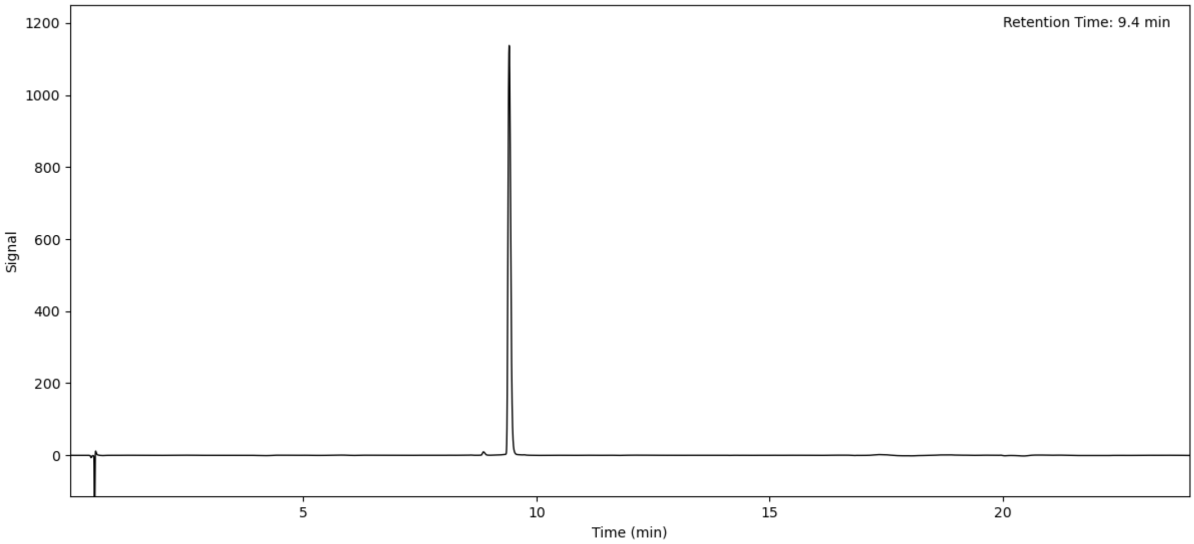

Peptide 7

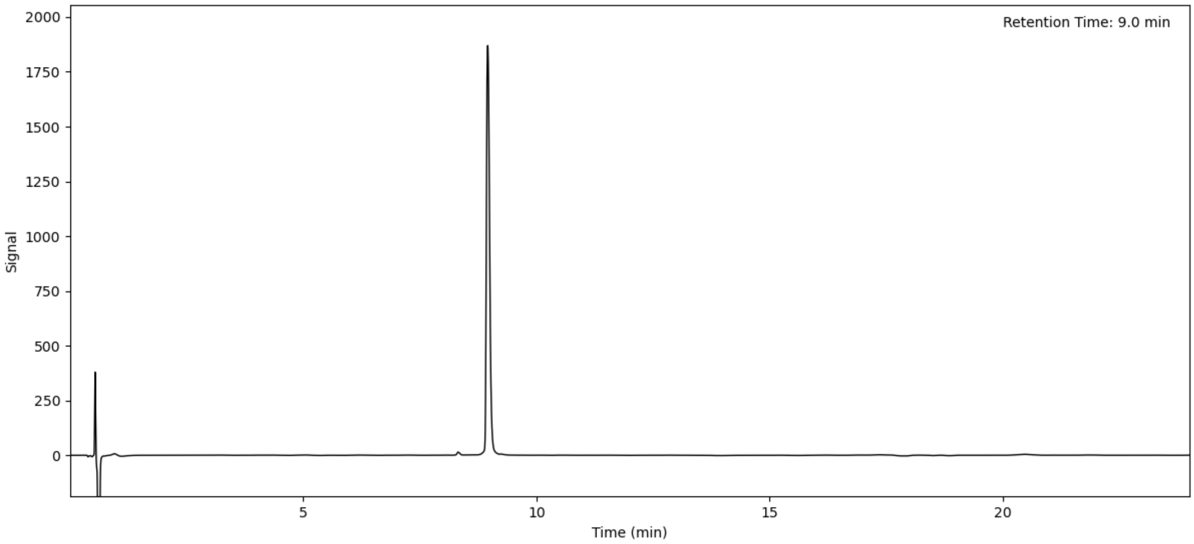

Peptide 8

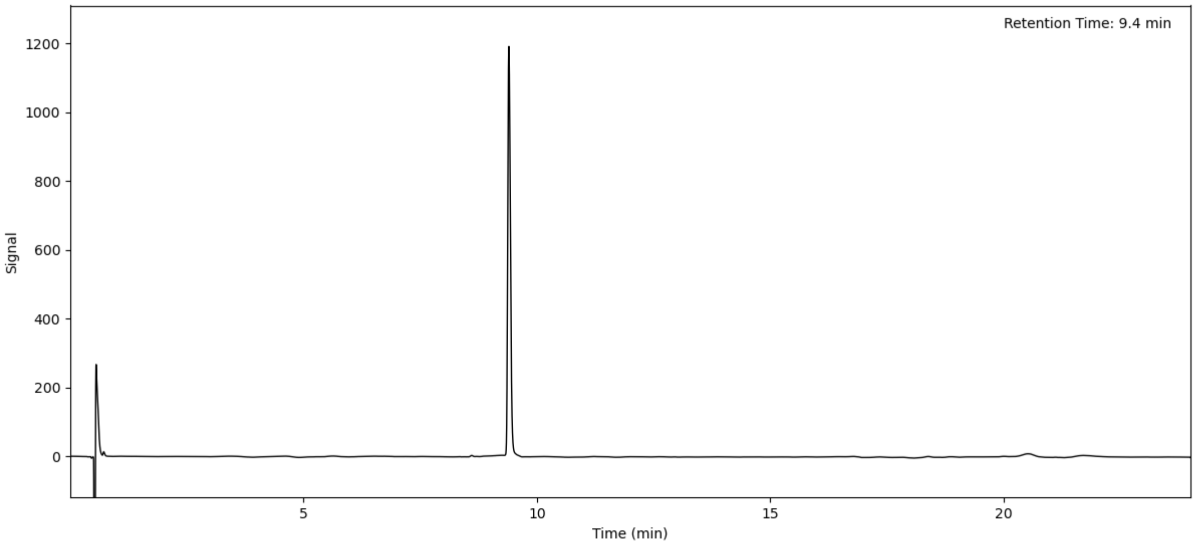

Peptide 9

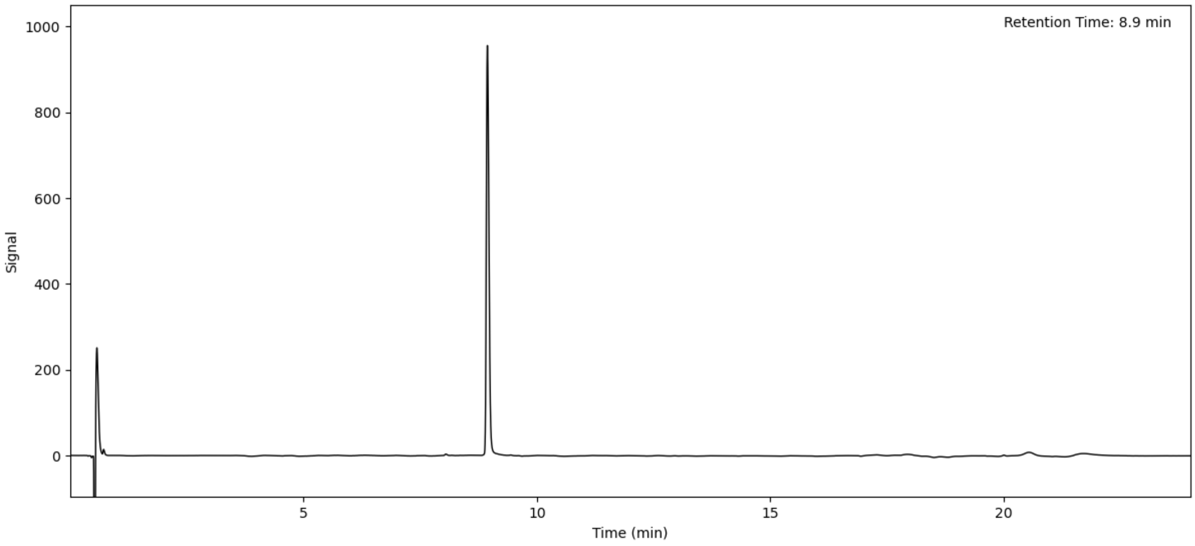

Peptide 10

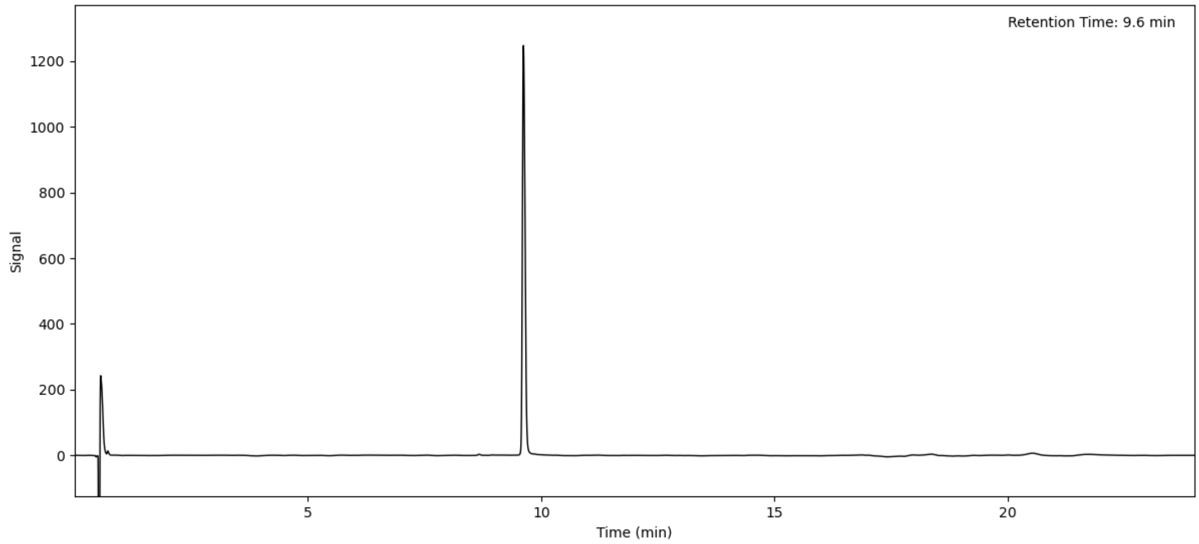

Peptide 11

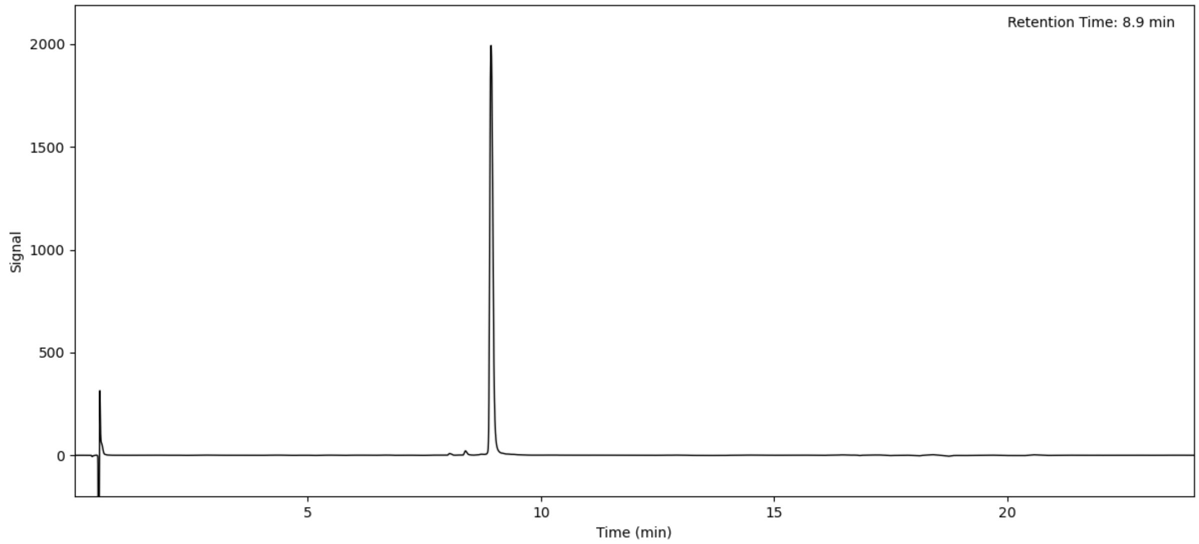

Peptide 12

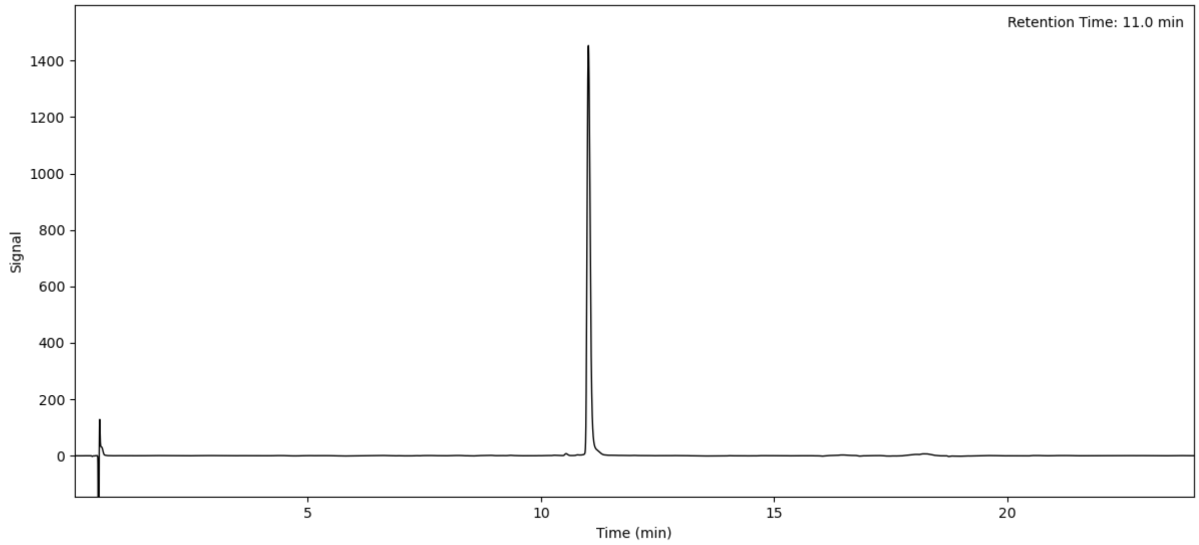

Peptide 13

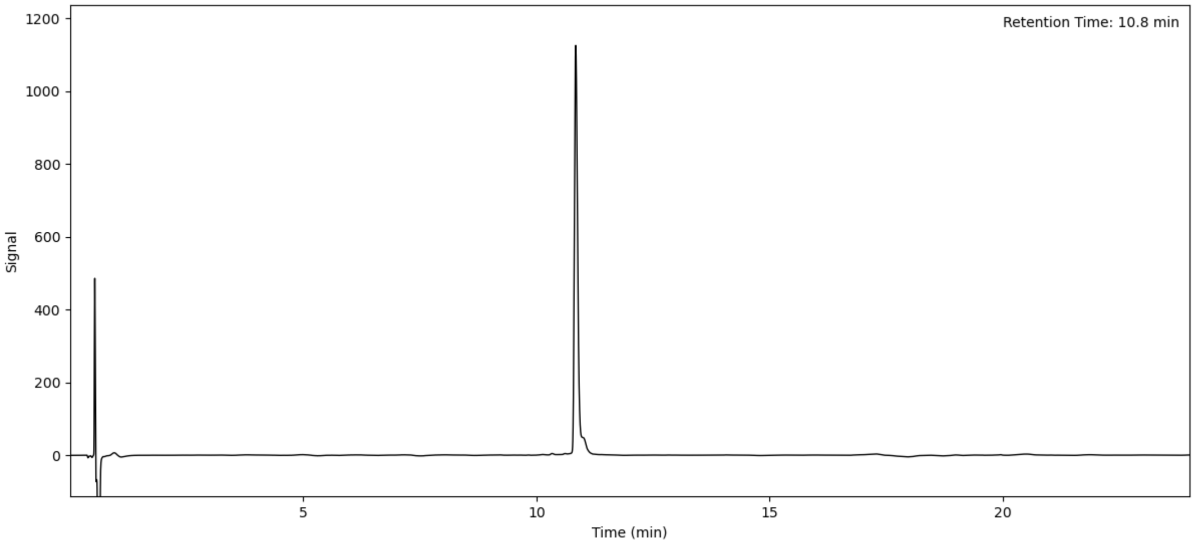

Peptide 14

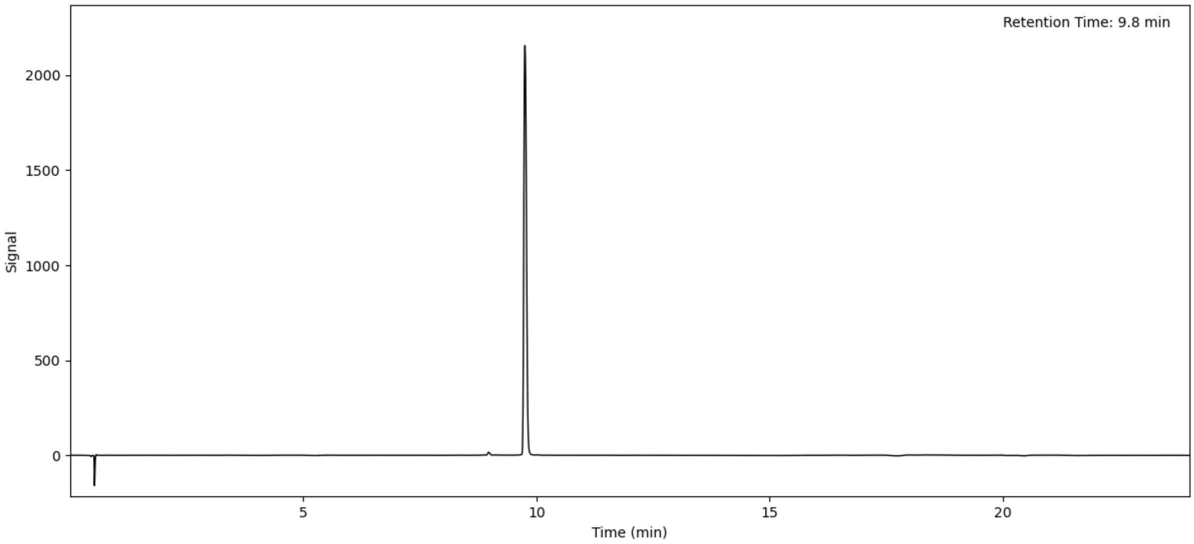

Peptide 15

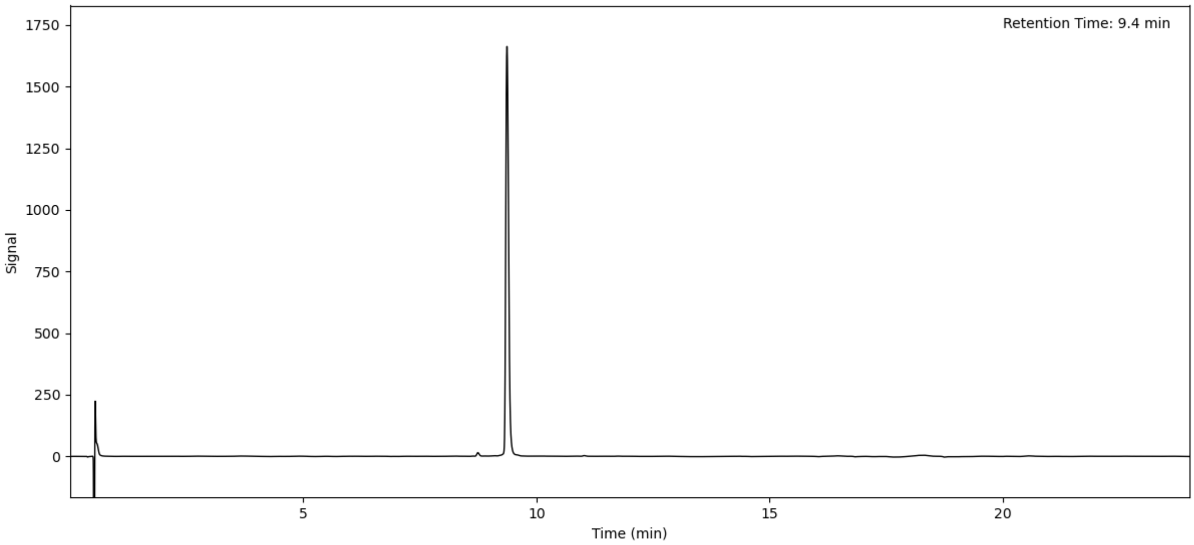

Peptide 16

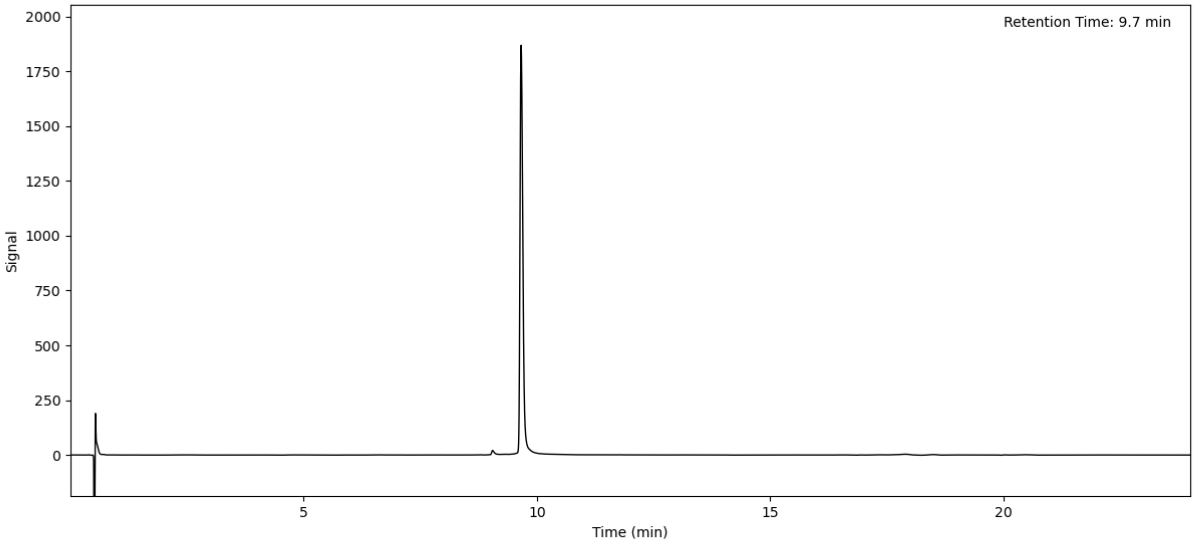

Peptide 17

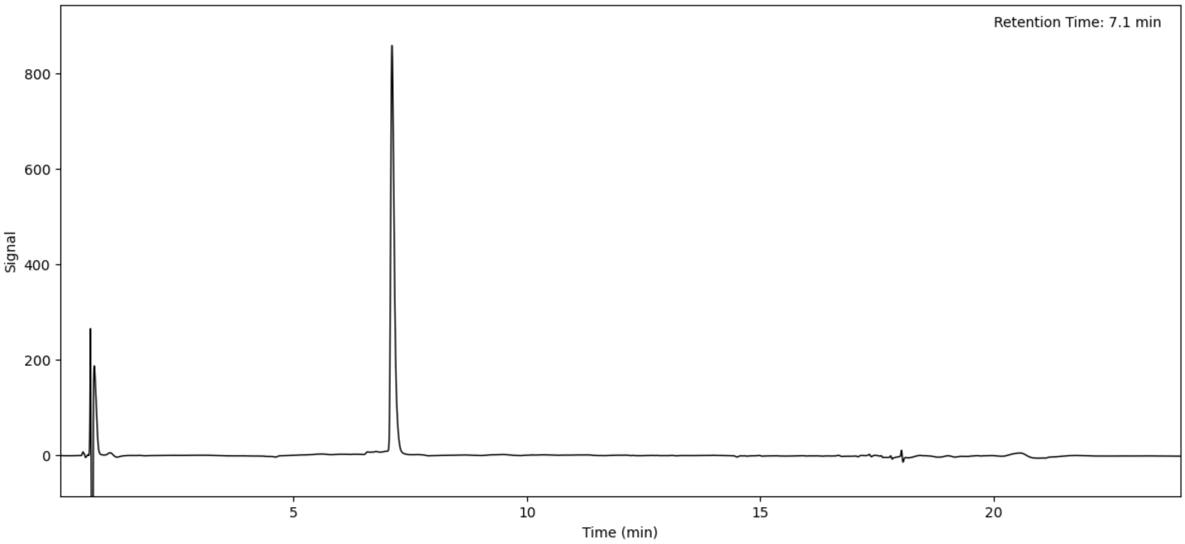

Peptide 18

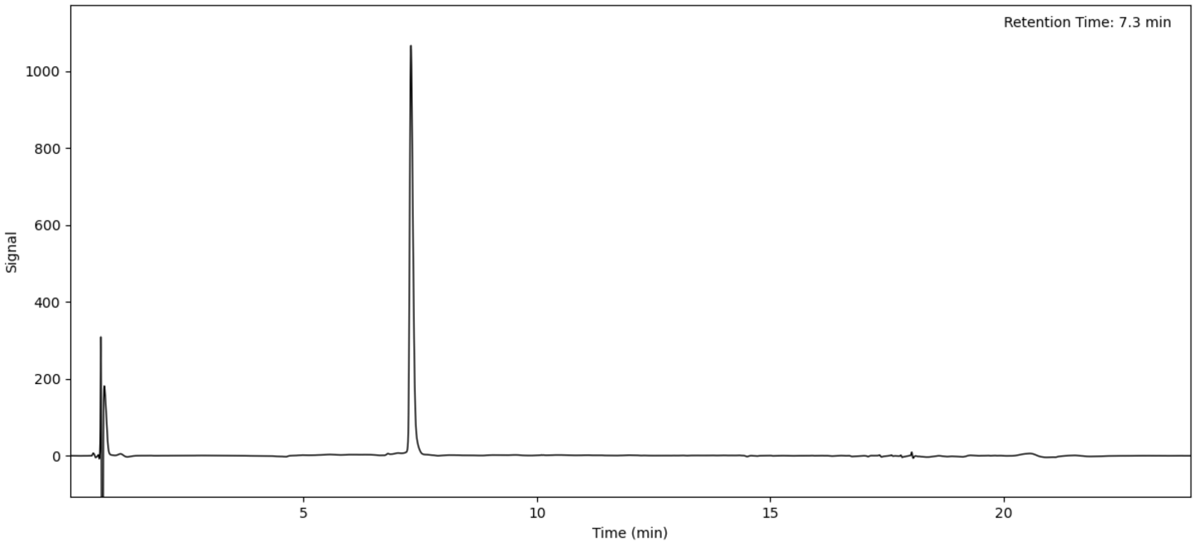

Peptide 19

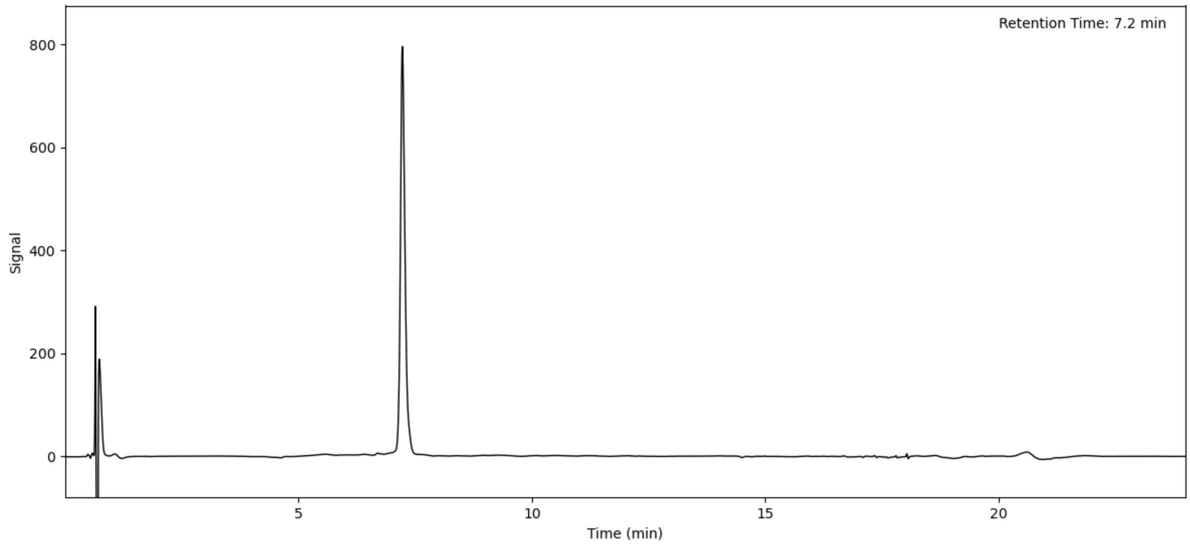

Peptide 20

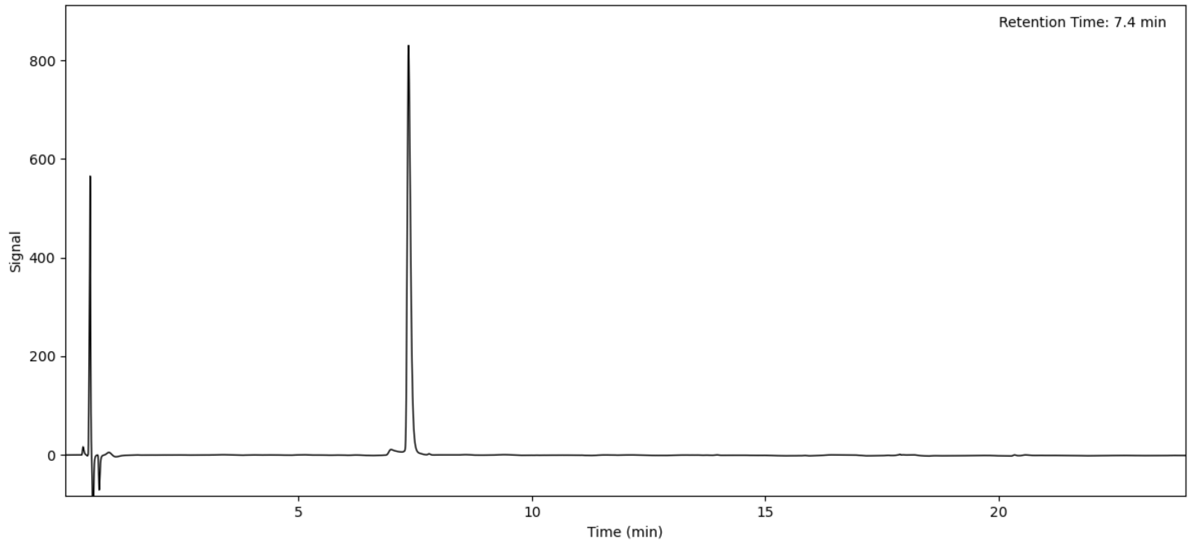

Peptide 21

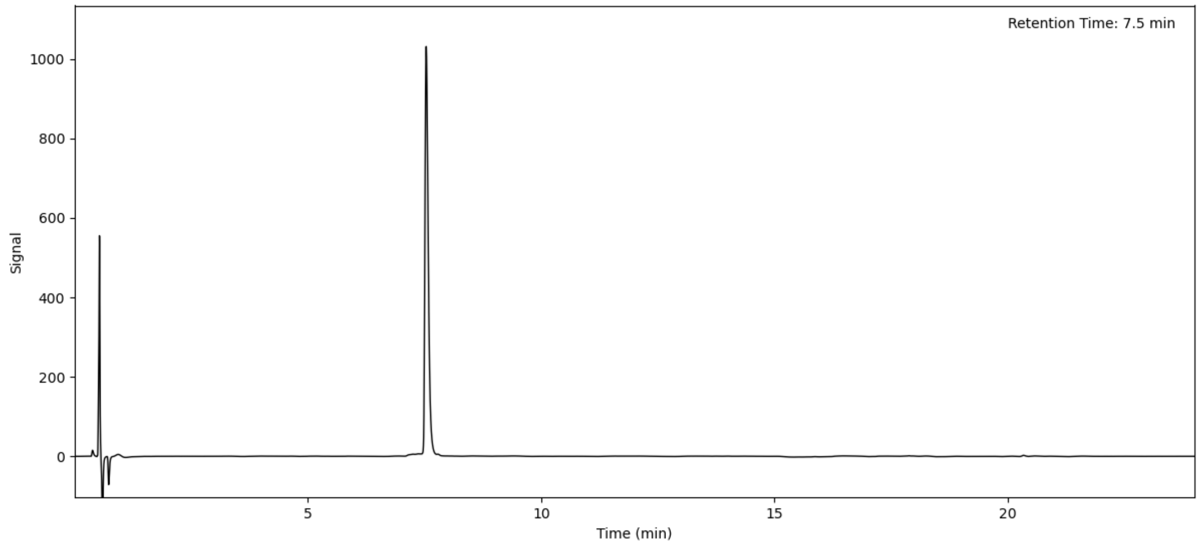

Peptide 22

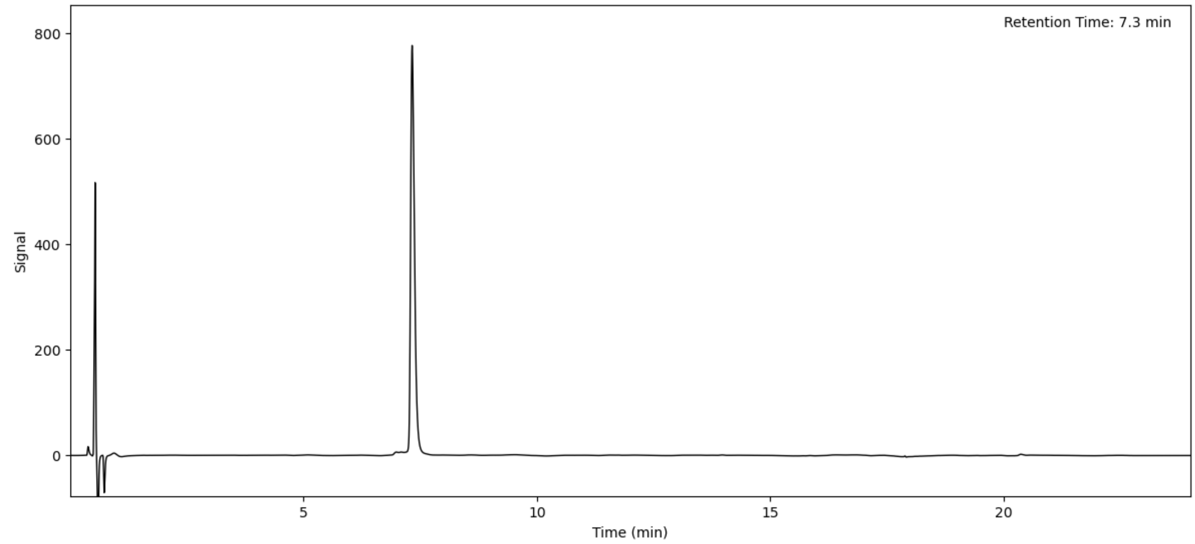

Peptide 23

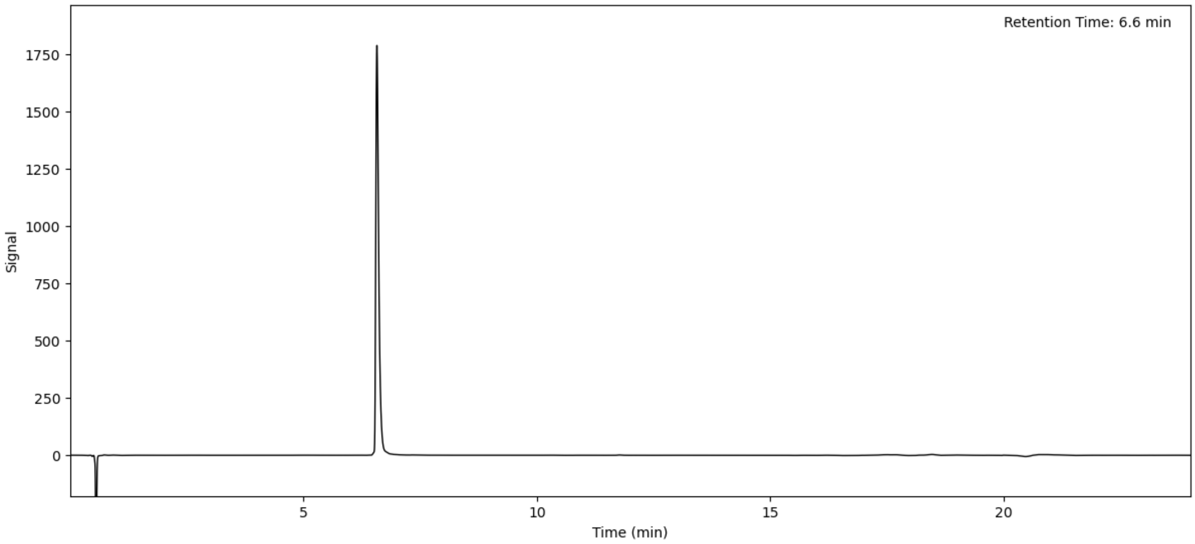

Peptide 24

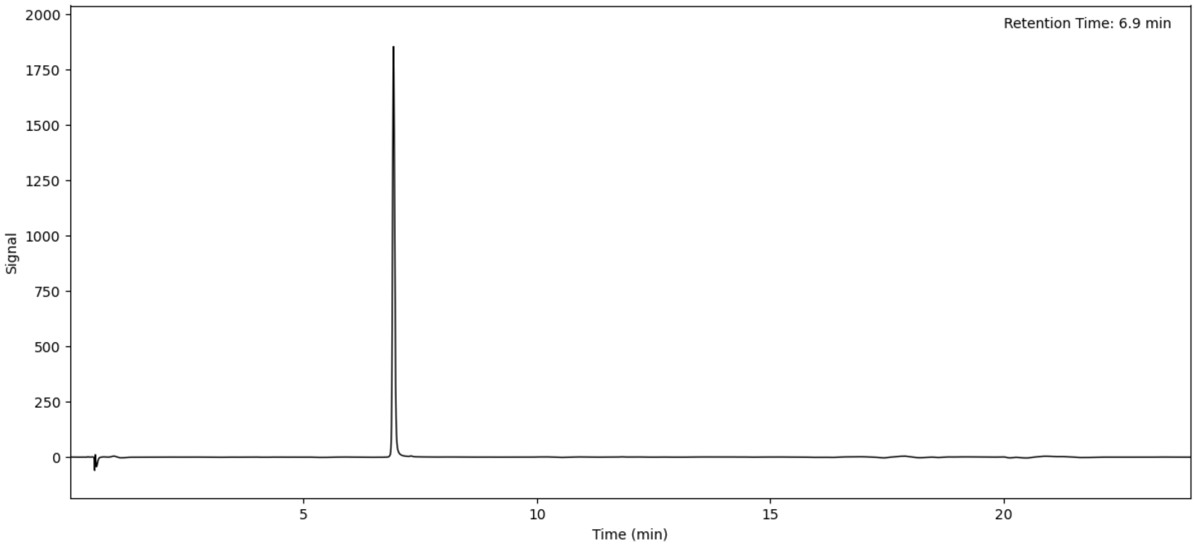

Peptide 25

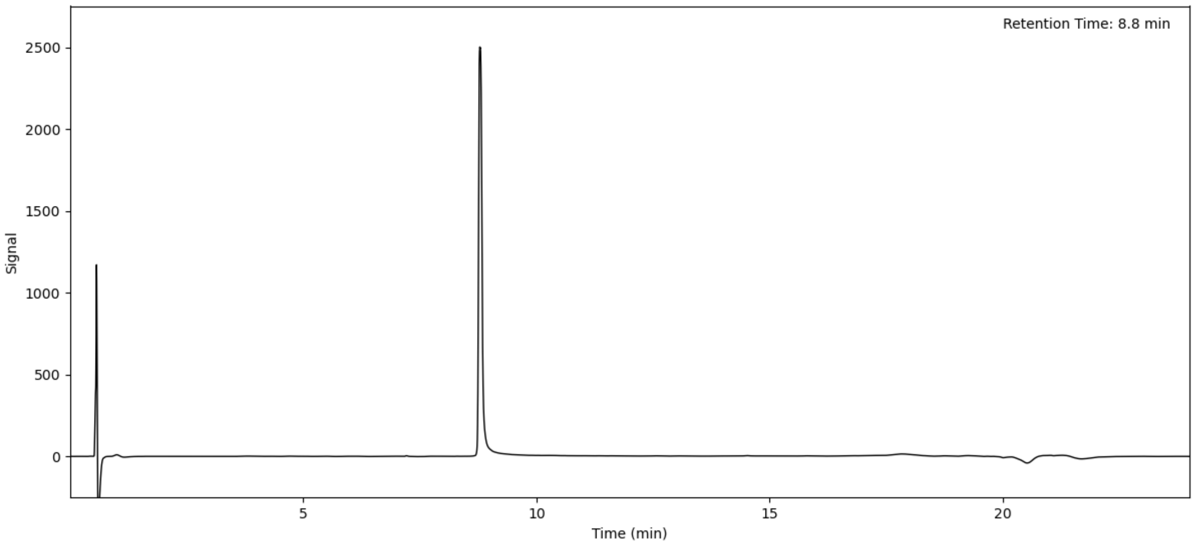

Peptide 26

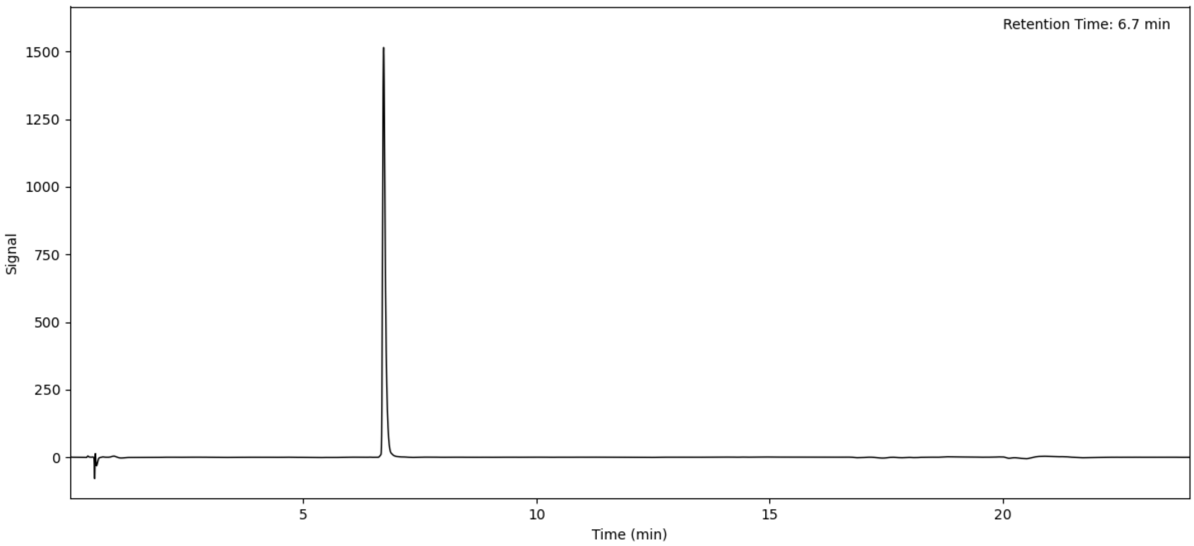

Peptide 27

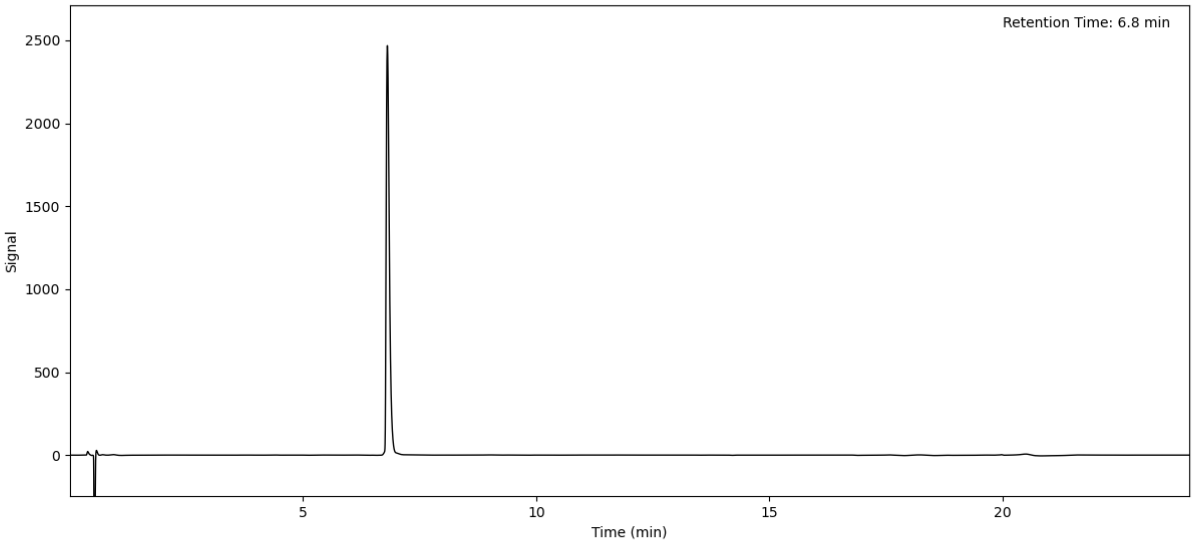

Peptide 28

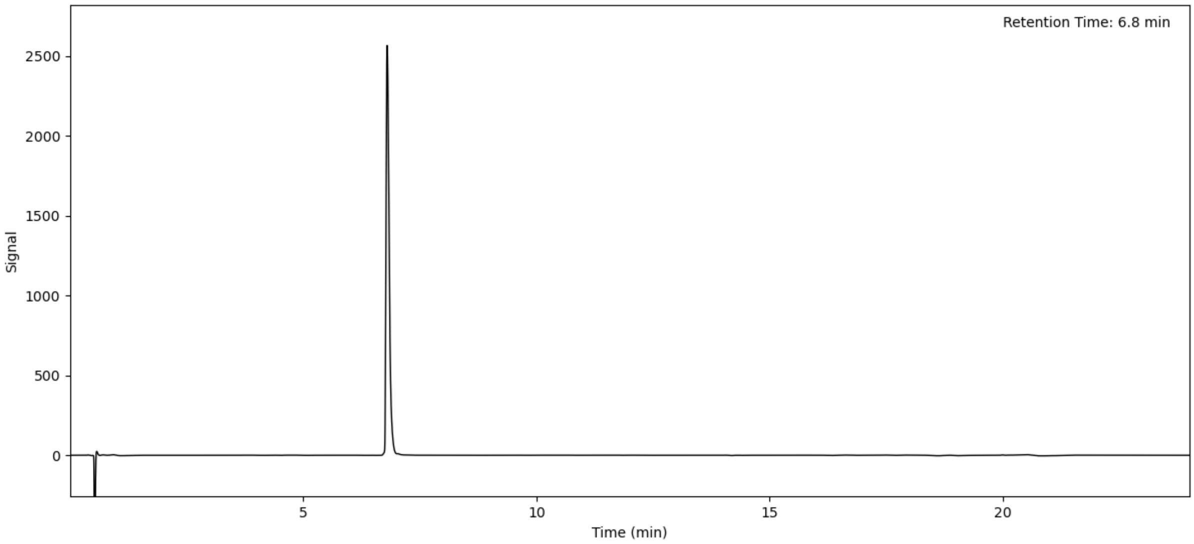

Peptide 29

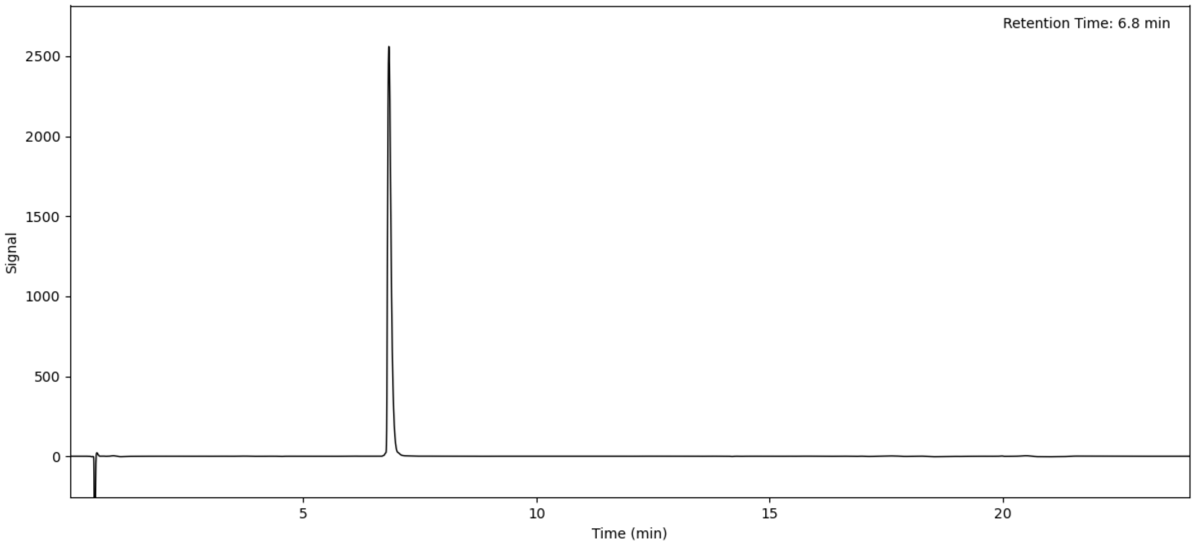

Peptide 30

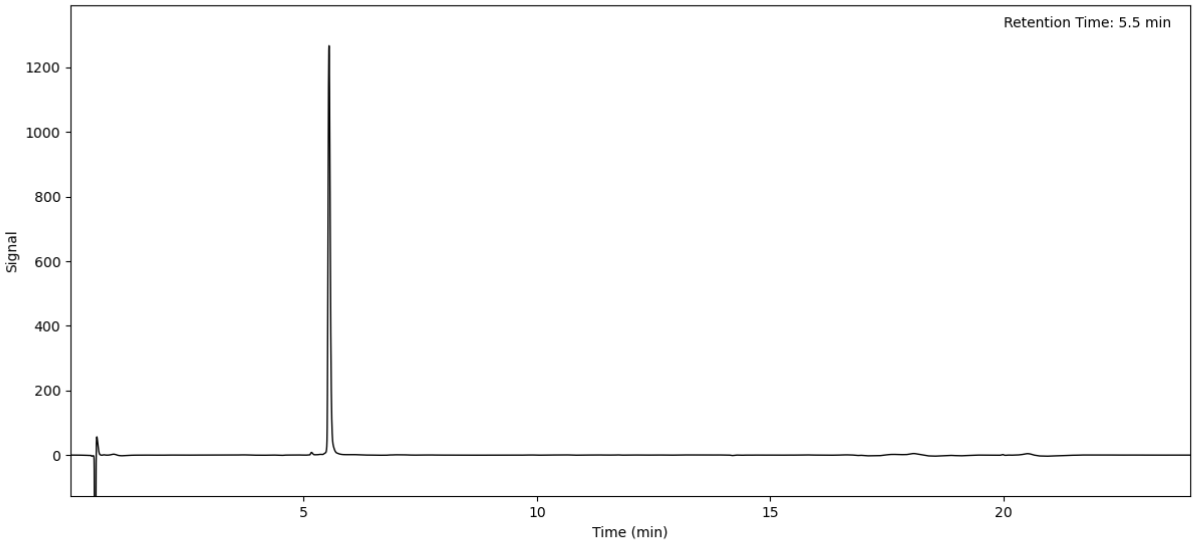

Peptide 31

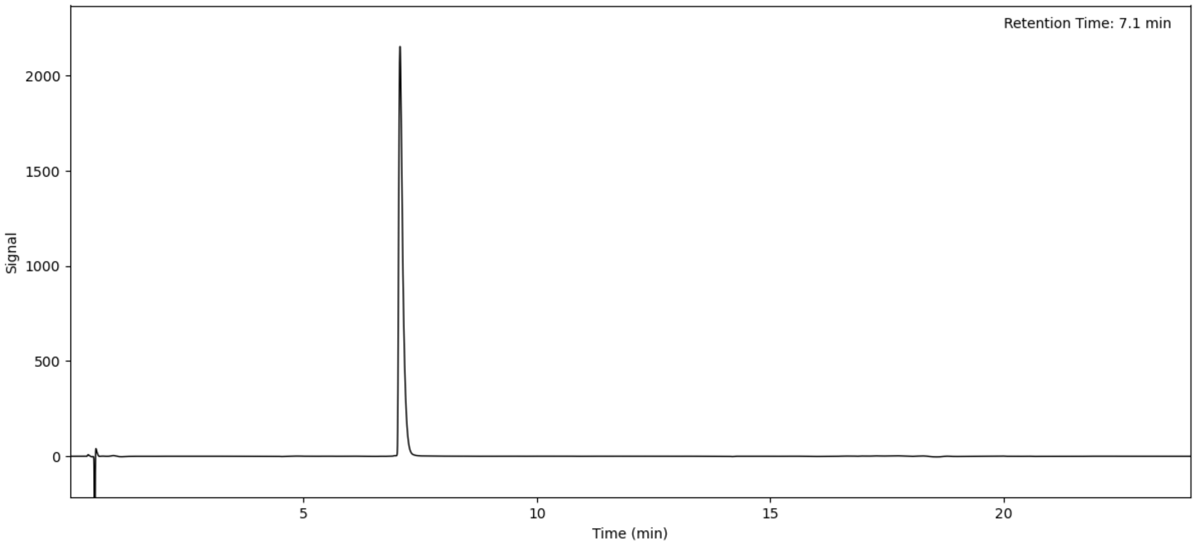

Peptide 32

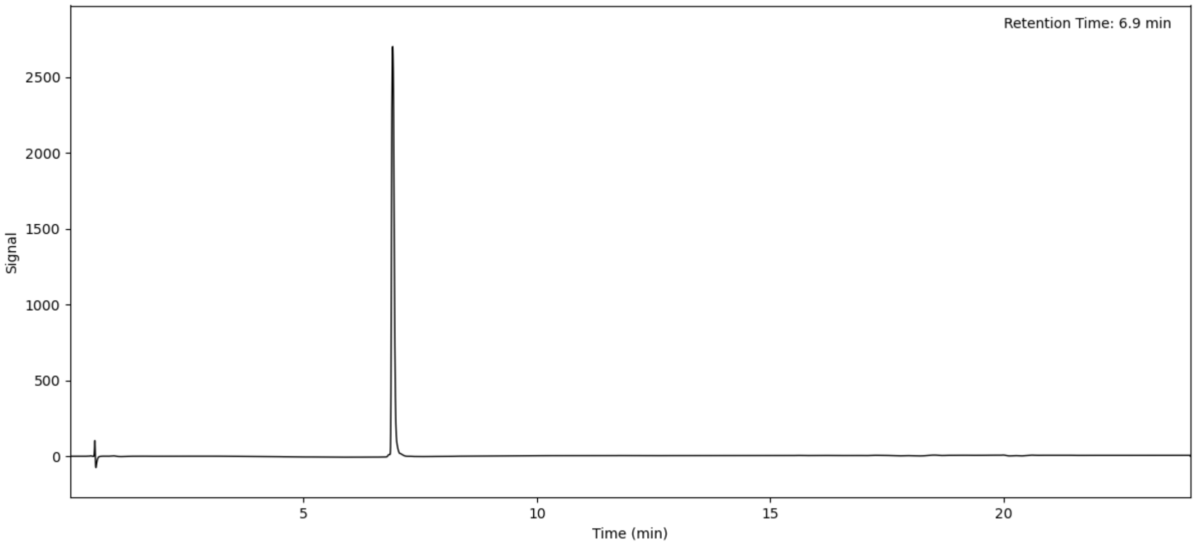

Peptide 33

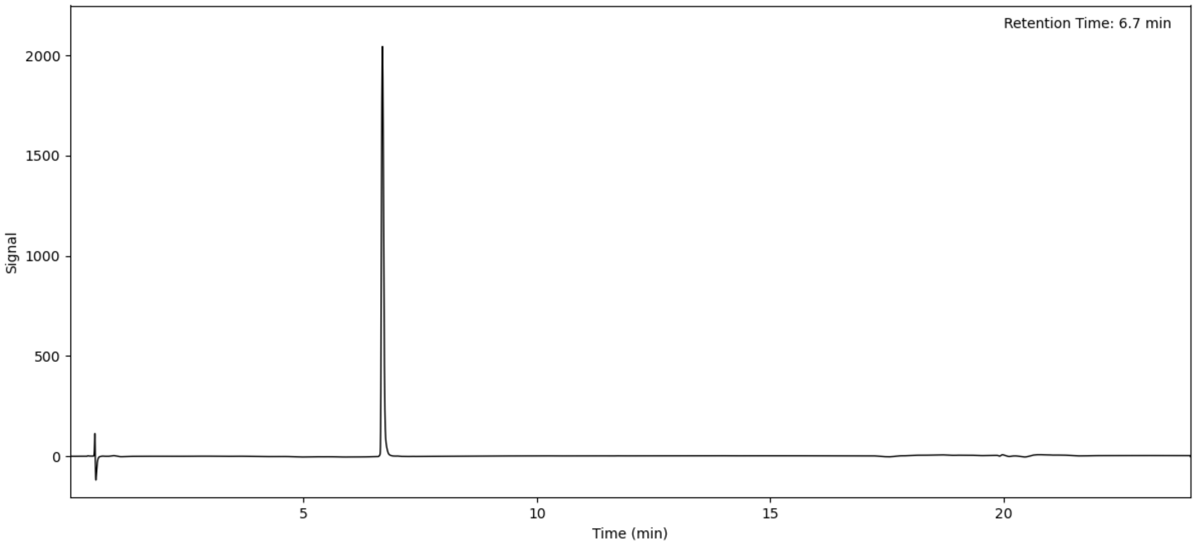

Peptide 34

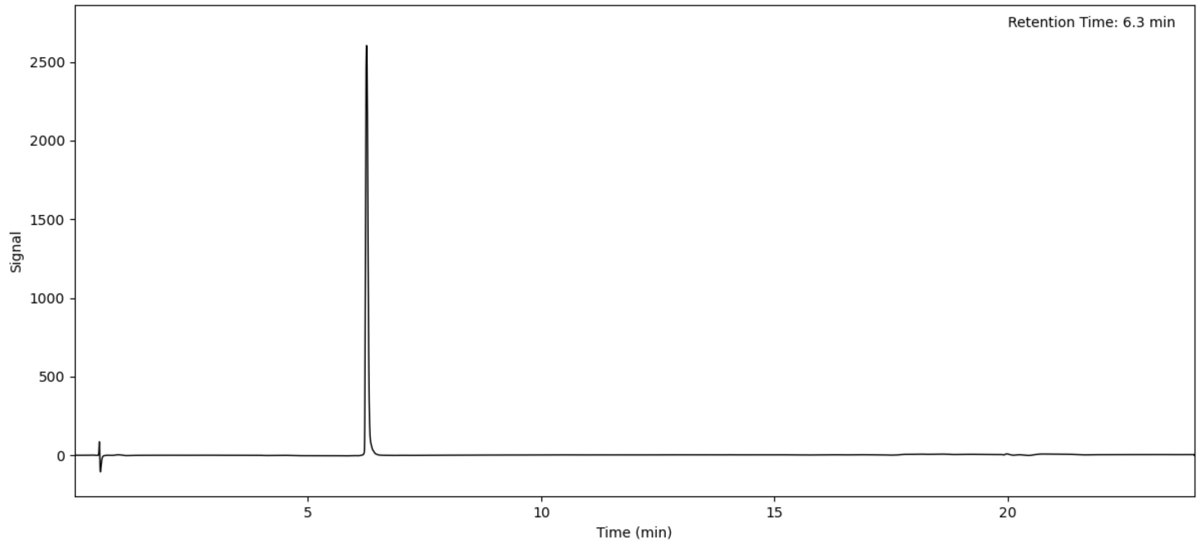

Peptide 35

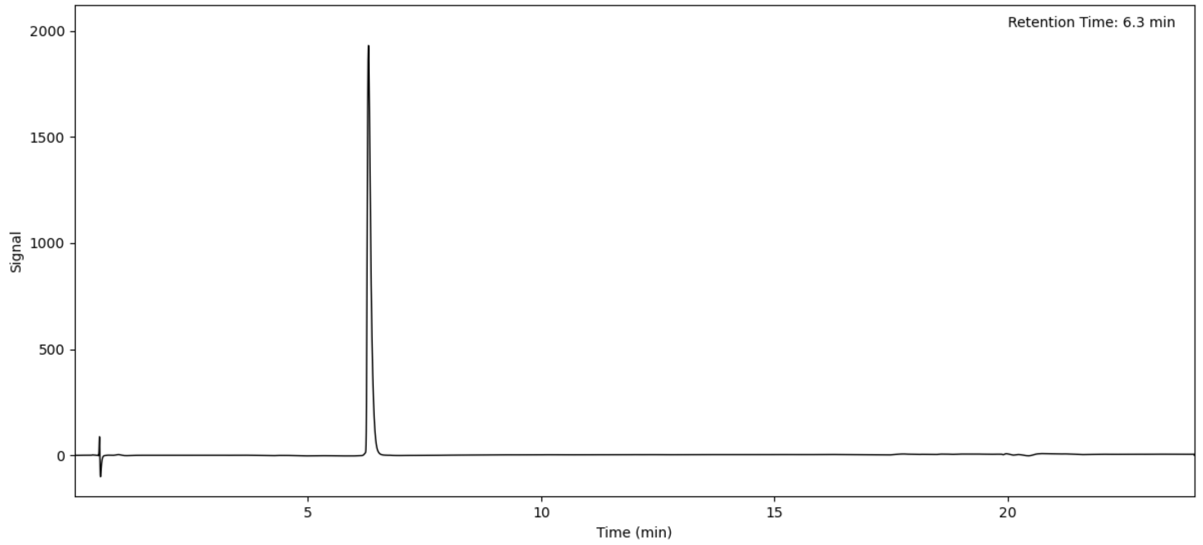

Peptide 36

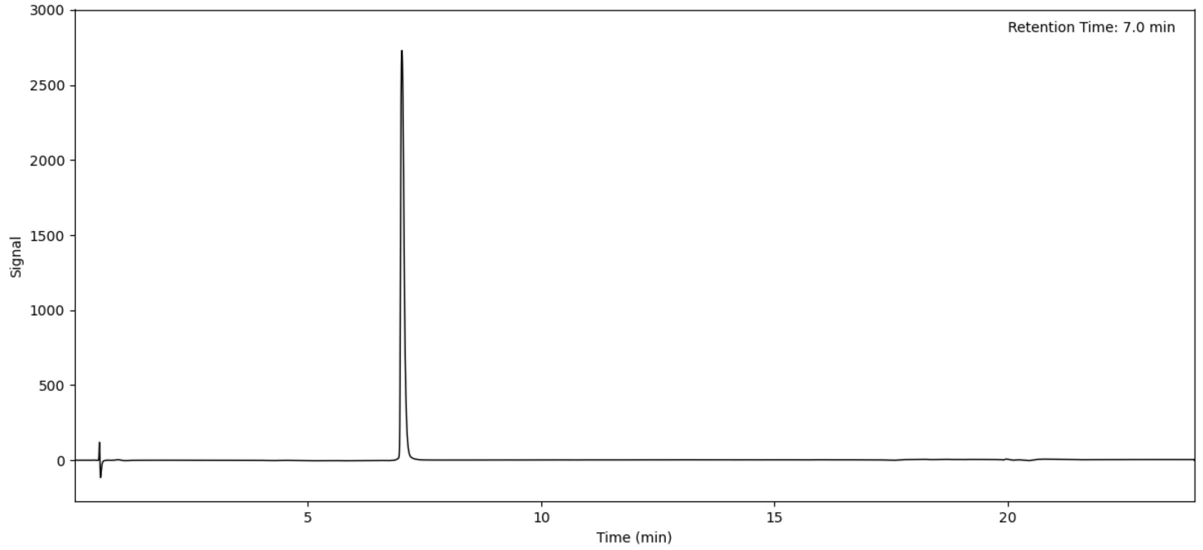

Peptide 37

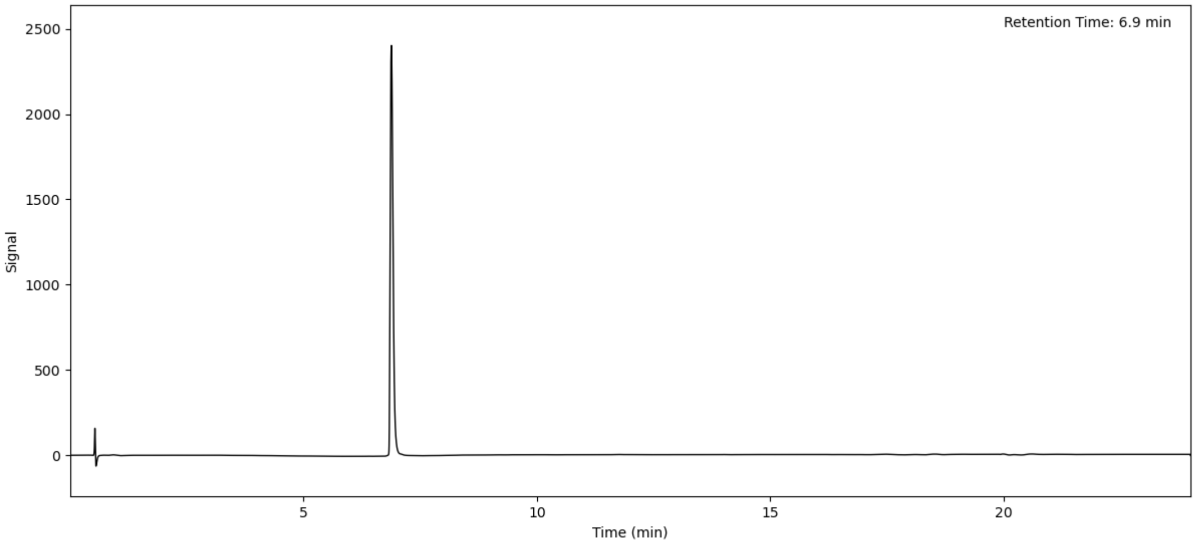

Peptide S1

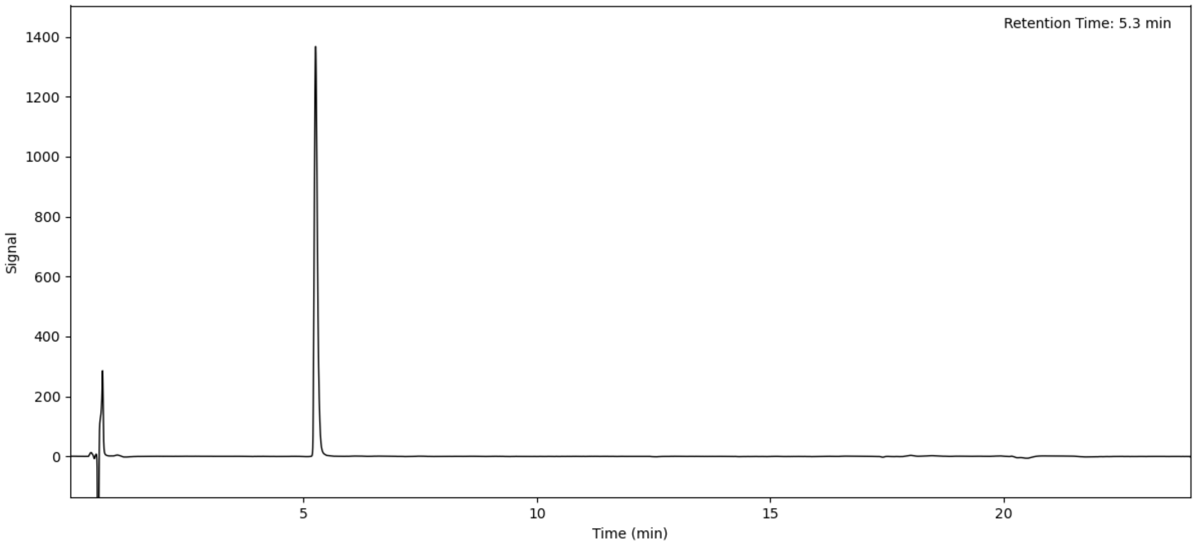

References

[1] M. Amblard, J.-A. Fehrentz, J. Martinez, G. Subra, *Mol. Biotechnol.* **2006**, *33*, 239–254.

[2] R. Subirós-Funosas, A. El-Faham, F. Albericio, *Biopolymers* **2012**, *98*, 89–97.

[3] G. Young, N. Hundt, D. Cole, A. Fineberg, J. Andrecka, A. Tyler, A. Olerinyova, A. Ansari, E. G. Marklund, M. P. Collier, S. A. Chandler, O. Tkachenko, J. Allen, M. Crispin, N. Billington, Y. Takagi, J. R. Sellers, C. Eichmann, P. Selenko, L. Frey, R. Riek, M. R. Galpin, W. B. Struwe, J. L. P. Benesch, P. Kukura, *Science (80-.).* **2018**, *360*, 423–427.

[4] D. Cole, G. Young, A. Weigel, A. Sebesta, P. Kukura, *ACS Photonics* **2017**, *4*, 211–216.

[5] S. Antonysamy, Z. Bonday, R. M. Campbell, B. Doyle, Z. Druzina, T. Gheyi, B. Han, L. N. Jungheim, Y. Qian, C. Rauch, M. Russell, J. M. Sauder, S. R. Wasserman, K. Weichert, F. S. Willard, A. Zhang, S. Emtage, *Proc. Natl. Acad. Sci. U. S. A.* **2012**, *109*, 17960–17965.

[6] K. Hsiao, H. Zegzouti, S. A. Goueli, *Epigenomics* **2016**, DOI 10.2217/epi.15.113.

[7] G. Winter, *J. Appl. Crystallogr.* **2010**, *43*, 186–190.

[8] G. Winter, D. G. Waterman, J. M. Parkhurst, A. S. Brewster, R. J. Gildea, M. Gerstel, L. Fuentes-Montero, M. Vollmar, T. Michels-Clark, I. D. Young, N. K. Sauter, G. Evans, *Acta Crystallogr. Sect. D Struct. Biol.* **2018**, *74*, 85–97.

[9] A. J. McCoy, R. W. Grosse-Kunstleve, P. D. Adams, M. D. Winn, L. C. Storoni, R. J. Read, *J. Appl. Crystallogr.* **2007**, *40*, 658–674.

[10] P. V. Afonine, R. W. Grosse-Kunstleve, N. Echols, J. J. Headd, N. W. Moriarty, M. Mustyakimov, T. C. Terwilliger, A. Urzhumtsev, P. H. Zwart, P. D. Adams, *Acta Crystallogr. Sect. D Biol. Crystallogr.* **2012**, *68*, 352–367.

[11] P. Emsley, B. Lohkamp, W. G. Scott, K. Cowtan, *Acta Crystallogr. Sect. D Biol. Crystallogr.* **2010**, *66*, 486–501.

[12] M. H. M. Olsson, C. R. SØndergaard, M. Rostkowski, J. H. Jensen, *J. Chem. Theory Comput.* **2011**, *7*, 525–537.

[13] W. L. Jorgensen, J. Tirado-Rives, *J. Am. Chem. Soc.* **1988**, *110*, 1657–1666.

[14] R. A. Friesner, J. L. Banks, R. B. Murphy, T. A. Halgren, J. J. Klicic, D. T. Mainz, M. P. Repasky, E. H. Knoll, M. Shelley, J. K. Perry, D. E. Shaw, P. Francis, P. S. Shenkin, *J. Med. Chem.* **2004**, *47*, 1739–1749.

[15] T. A. Halgren, R. B. Murphy, R. A. Friesner, H. S. Beard, L. L. Frye, W. T. Pollard, J. L. Banks, *J. Med. Chem.* **2004**, *47*, 1750–1759.

[16] R. A. Friesner, R. B. Murphy, M. P. Repasky, L. L. Frye, J. R. Greenwood, T. A. Halgren, P. C. Sanschagrin, D. T. Mainz, *J. Med. Chem.* **2006**, *49*, 6177–6196.

[17] D. M. Krüger, H. Gohlke, *Nucleic Acids Res.* **2010**, *38*, 480–486.
